# Generation, annotation, and analysis of an extensive *Aspergillus niger *EST collection

**DOI:** 10.1186/1471-2180-6-7

**Published:** 2006-02-02

**Authors:** Natalia Semova, Reginald Storms, Tricia John, Pascale Gaudet, Peter Ulycznyj, Xiang Jia Min, Jian Sun, Greg Butler, Adrian Tsang

**Affiliations:** 1 Centre for Structural and Functional Genomics, Concordia University, Montreal, Canada; 2 Department of Biology, Concordia University, Montreal, Canada; 3 Department of Computer Science and Software Engineering, Concordia University, Montreal, Canada; 4 Northwestern University, 676 N. St. Clair Street, Chicago, IL 60611

## Abstract

**Background:**

*Aspergillus niger*, a saprophyte commonly found on decaying vegetation, is widely used and studied for industrial purposes. Despite its place as one of the most important organisms for commercial applications, the lack of available information about its genetic makeup limits research with this filamentous fungus.

**Results:**

We present here the analysis of 12,820 expressed sequence tags (ESTs) generated from *A. niger *cultured under seven different growth conditions. These ESTs identify about 5,108 genes of which 44.5% code for proteins sharing similarity (E ≤ 1e ^-5^) with GenBank entries of known function, 38% code for proteins that only share similarity with GenBank entries of unknown function and 17.5% encode proteins that do not have a GenBank homolog. Using the Gene Ontology hierarchy, we present a first classification of the *A. niger *proteins encoded by these genes and compare its protein repertoire with other well-studied fungal species. We have established a searchable web-based database that includes the EST and derived contig sequences and their annotation. Details about this project and access to the annotated *A. niger *database are available.

**Conclusion:**

This EST collection and its annotation provide a significant resource for fundamental and applied research with *A. niger*. The gene set identified in this manuscript will be highly useful in the annotation of the genome sequence of *A. niger*, the genes described in the manuscript, especially those encoding hydrolytic enzymes will provide a valuable source for researchers interested in enzyme properties and applications.

## Background

Members of the genus *Aspergillus*, including *Aspergillus niger*, are distributed worldwide and are commonly present on decaying plant debris. These saprophytes degrade the complex molecules in plant cell materials by secreting an extensive assortment of hydrolytic enzymes [1]. Since *A. niger *grows on organic matter over a wide range of temperature, 6–47°C, and pH, 1.4–9.8 [2], this fungus produces enzymes that are active in diverse environmental conditions. Indeed, many enzymes produced by this fungus have already found application in the food, beverage, textile, agriculture, and paper and pulp industries [1,3]. *A. niger *is also widely used in the manufacture of organic acids including citric, gluconic and fumaric acids [4,5]. Importantly, citric acid and many enzymes produced in *A. niger *have received 'generally regarded as safe' or GRAS status by the United States Food and Drug Administration (FDA), and can therefore, be safely used for agro-food applications [2].

*Aspergillus niger*, with its long history of use for various industrial applications and the ability to efficiently produce native proteins, is an attractive host for the production of heterologous proteins [6]. The commercial production of heterologous proteins using *A. niger *started when Genencor International (San Francisco) produced bovine chymosin in *A. niger *[7] and received US FDA approval for its application in cheese making. *A. niger *has subsequently been used as an expression host to produce commercially viable levels of many heterologous proteins, including; human cytokine interleukin -6 (*IL-6*) [8], *Phanerochaete chrysosporium *manganese peroxidase (*MnP*) [9], barley alpha-amylase [10], porcine pancreatic prophospholipase A2 (*proPLA2*) [11], and correctly assembled human immunoglobulins [12].

*Aspergillus niger *is presently one of the most important organisms used in biotechnology. Reflecting this, there are 784 genomic DNA and mRNA sequence entries representing 379 unique genes available in GenBank databases (July 20, 2005 release). The identification of additional genes will enhance further efforts to increase the industrial utility of this organism. Analysis of EST sequences provides a cost-effective approach for gene discovery. Furthermore, EST-derived sequences facilitate genome sequence annotation through the identification of transcription unit boundaries, exon-intron junctions, and genes that lack sequence similarity with previously discovered genes. For these reasons, we initiated an *A. niger *EST-based gene discovery program. Using normalization methods to enrich for cDNA templates representing weakly expressed genes we identified 5,108 unique genes of which 44.5% encode proteins with significant similarity to GenBank entries that have at least a tentatively assigned function. Using the Gene Ontology hierarchy [13], we present a classification of the proteins encoded by these *A. niger *genes and compare its protein repertoire with other well-studied fungal species. Our annotated *A. niger *EST collection is available at our website [14].

## Results and discussion

### Library normalization and subtraction

**Figure 1:**
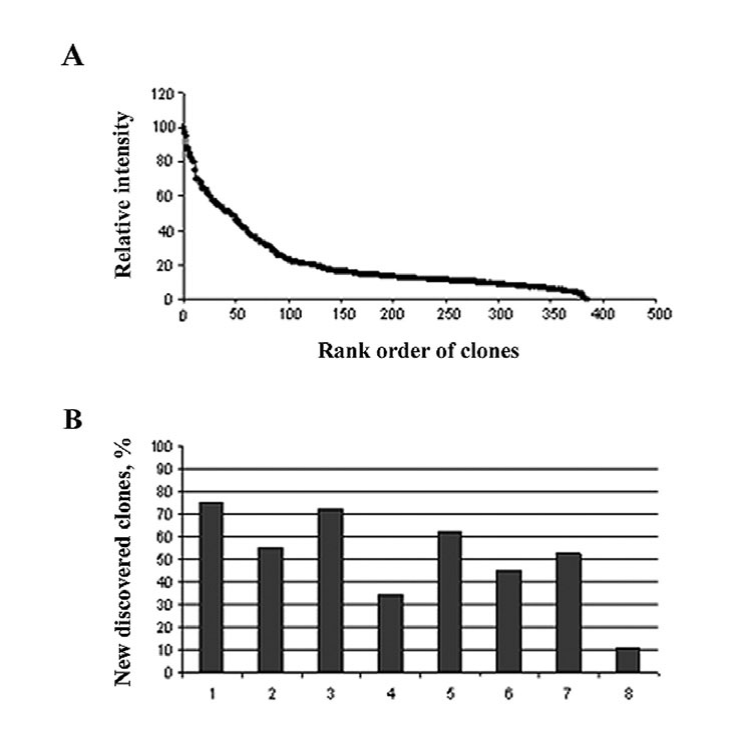
**Virtual normalization and direct subtraction**. A) Relative signal intensity of clones, determined as a ratio of the signal intensity of each individual clone versus the maximum signal intensity present on the array. Signal intensities for the colonies derived from a single 384 well microplate are displayed as a function of relative colony signal strength. B) Proportion of unique ESTs obtained at various stages of the gene discovery process. 1: Sequencing of the first 192 randomly selected clones, 2: Sequencing of the last 192 clones of the 1920 randomly selected clones. 3: Sequencing of the first 192 clones obtained by virtual normalization. 4: Sequencing of the last 192 clones from the 2,304 clones obtained by virtual subtraction. 5: Sequencing of the first 192 clones selected after virtual normalization and direct subtraction, first round. 6: Sequencing of the last 192 clones selected after both virtual normalization and direct subtraction, first round. 7: Sequencing of the first 192 clones selected after virtual normalization and direct subtraction, second round. 8: Sequencing of the last 192 clones from the 10,738 clones selected after virtual normalization and direct subtraction, second round.

A major challenge confronting EST-based gene discovery programs is differential mRNA abundance. Usually, a few hundred highly and moderately expressed genes produce more than half of the cellular mRNA molecules, whereas several thousand genes account for the remaining mRNA mass [15]. Sequencing randomly selected clones from standard cDNA libraries therefore inefficiently identifies rare transcripts, owing to the repeated occurrence of moderately and highly abundant cDNA species. We employed virtual subtraction and direct subtraction to enhance the number of unique genes identified. The virtual subtraction method [16] classifies cDNA clones according to the abundance of the mRNAs they represent (Figure 1A). The direct subtraction method removes previously identified cDNA clones from the gene discovery pipeline. We initiated this EST-based gene discovery program by sequencing 2,000 randomly selected clones. Next, we sequenced 2,304 of the low intensity clones identified by virtual subtraction. Finally, we sequenced 10,738 clones that gave very low hybridization signals when subjected to both virtual and direct subtraction.

Figure 1B presents the gene discovery rates obtained while sequencing the randomly selected clones, the clones selected following virtual subtraction, and the clones selected following virtual and direct subtraction. We obtained 5,202 singleton and contig sequences after processing 12,820 high quality EST sequences (Table 1). This means that we identified roughly one gene for every 2.5 EST sequences. This result compares favorably with the results obtained by some other large-scale EST projects of lower eukaryotes. For instance, a *Neurospora crassa *project produced 20,019 ESTs and identified 1,431 genes [17] for a gene discovery rate of one gene for every 14 EST sequences, and a *Dictyostelium discoideum *gene discovery project that generated 26,954 ESTs identified 5,381 unigenes for a gene discovery rate of one gene for every 5 EST sequences [18].

### Contig assembling and analysis of *A. niger *ESTs

We submitted the 12,820 high quality ESTs to GenBank [GenBank: DR697868 – GenBank: DR710686]. Table 1 shows that the individual sequencing reads contained 400–800 nucleotides of high-quality sequence. The EST assembly produced by phrap [19] yielded 5,202 unisequences that included 2,183 singletonsand 3,019 contigs. Following assembly, we used BLASTN to cluster the closely related singletons and contigs. Clustering assembled 168 of the 5,202 phrap unisequences into 74 clusters, each containing 2–4 sequences. Manually confirmed ClustalW alignments showed that 56 clusters were generated by assembling alternatively spliced derivatives of 117 phrap unisequences. Taking into account the 74 clusters assembled from multiple unisequences, the 12,820 ESTs generated 5,108 clusters. The clusters predicted to have arisen through alternative splicing are available in Additional file 1. Prior to submission of our EST sequences, we found 784 *A. niger *genomic DNA and cDNA-derived sequence entries in the GenBank database (June 22, 2005 release). These entries formed 379 unique genes. BLASTN analysis showed that 252 of the phrap unisequences aligned with at least one of the *A. niger *GenBank entries (alignment length >50, identity >95%). Therefore, this study identified about 4,856 new *A. niger *genes. The results from our EST sequencing, contig assembly and clustering analysis are summarized in Table 1.

### Comparative analysis of the phrap unisequences

We attempted to determine the putative function of the set of 5,202 phrap unisequences by searching for homologs in the GenBank non-redundant protein database using BLASTX (Table 2). Setting the BLASTX cutoff value at E = 1e ^-5^, about 83% of these sequences display similarity to at least one GenBank entry, 44.5% to genes of known function and 38% to genes of unknown function. The remaining sequences, 17 %, code for proteins that lack similarity with any GenBank entry.

We also compared the proteins encoded by these sequences with the proteins predicted from the completely sequenced genomes of three Ascomycetes, *Saccharomyces cerevisiae *[20], *Aspergillus nidulans *and *Neurospora crassa *[21], and one Basidiomycete, the white rot fungus *Phanerochaete chrysosporium *[22]. As expected, the highest degree of similarity (BLASTX alignments with E values ≤ e^-30^) is with *A. nidulans*, where 64% of these *A. niger *unisequences encode proteins that have *A. nidulans *homologs (Table 2). Nonetheless, almost 20% of the *A. niger *genes did not have a homolog (E > e^-5^) in *A. nidulans*.

Although the Sordariomycetes, which include *N. crassa*, and the Eurotiomycetes, which include the Aspergilli, diverged about 670 million years (Myr) ago [23], over 43% of the predicted *A. niger *proteins are highly similar (E ≤ e^-30^) to *N. crassa *predicted proteins. For the more distantly related Saccharomycotinna *S. cerevisiae *and Hymenomycete *P. chrysosporium*, which diverged from the Eurotiomycetes lineage about 1,090 and 1,210 Myr ago, respectively [23], only 21% and 25% of the *A. niger *predicted proteins had highly similar homologs (E ≤ e^-30^).

### Functional classification of genes based on Gene Ontology terms

**Figure 2:**
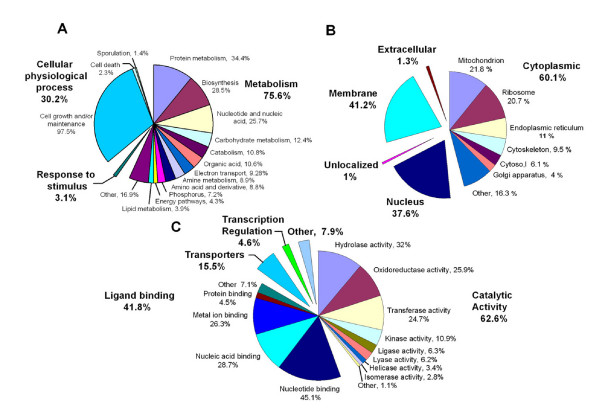
**GO mappings for the *A. niger *proteins**. Relative representation of GO mappings for the proteins coded for by the unique set of *A. niger *singletons and contigs. A) Biological process; B) Cellular component; C) Molecular function. Note, because individual proteins can map to multiple GO categories, the sum of the GO mappings can exceed 100%.

The predicted *A. niger *protein products were assigned Gene Ontology (GO) classifiers based on BLASTX alignments (expected values of E ≤ e^-5^) generated by searching the GO annotated Swiss-Prot and TrEMBL databases. GO categories were assigned to 2,549 of the 5,202 predicted protein products. Figure 2 summarizes the resulting GO assignments, which are available in Additional file 2. More detailed annotations, including the BLAST alignments, Expect Values and BLAST Scores generated by searching the GenBank nr database are available online [14] and can be used to assess the reliability of functional predictions on a gene by gene basis.

We compared the distribution of GO classifiers obtained for the *A. niger *unisequences and the predicted genes of six fungal species (Table 3). The gene distribution in the main ontology categories was very similar across all seven species. However, the fission and budding yeasts have a higher proportion of genes in the "cell growth and/or maintenance" categories, 45.2% and 48.5%, than did the filamentous fungi, where the proportion ranged from 29.4% to 36.2%. Since we found no correlation between evolutionary distance and these differences, it seems likely that they reflect differences in gene number. The genomes of the five filamentous fungi encode 9,000–12,000 genes [24,25] whereas the fission and baker's yeast genomes have about 4,824 [26] and 6,335 [27] protein-coding genes, respectively. The much smaller number of genes present in these two yeast species suggests that they may have close to the minimum number of genes needed by a free-living eukaryotic cell [28].

### Identification of putative secreted proteins

*Aspergillus niger *is the source of a number of secreted proteins produced for various industrial applications. Gene Ontology mapping categorized only 15 of the predicted proteins as "extracellular" (Additional file 2 ). However, we were able to assign a GO component classifier to only 1,195 (23.4%) of the encoded proteins. To identify potential secreted proteins we used SignalP 3 [29] to search for proteins with a secretion signal. SignalP predicted that about 400 of the predicted proteins had a signal peptide (Additional file 3 ). Blast searches showed that 293 of these proteins were similar (E ≤ e^-5^)to at least one GenBank entry. The 27% of predicted proteins with a signal peptide that do not have a GenBank homolog is significantly higher that the 17.5% of predicted orphan proteins. The reason for these differences remains unknown although they may suggest that the fungal secretome is subject to rapid evolution.

### Characterization of secretion pathway proteins

Recent strategies for improving the efficiency of heterologous protein expression in *A. niger *have focused on molecular genetic manipulation of the secretory pathway. In some cases, these approaches have significantly increased the expression of selected heterologous proteins [30,31]. Using GO mappings and BLAST analysis we identified 118 genes that apparently participate in various steps of the protein secretion pathway (Additional file 4 ). Fifteen genes encode secretion-related ER chaperones, foldases and proteases; 77 encode putative proteins involved in protein transport, protein targeting and vesicle-mediated transport; and 26 code for proteins that are involved in secretion-related post-translational modifications. The *A. niger *genes identified in this study included all the previously identified secretion-related ER chaperones, foldases and quality control proteins: *bipA *(Asp84), *pdiA *(Asp734, Asp1902), *prpA *(Asp4188), *tigA *(Asp1020), *cybB *(Asp662), *clxA *(calnexin) (Asp1882), and *kexB *(kexin) (Asp177) [30-33].

Previous studies with *A. niger *identified five secretion-related GTPases belonging to the Ras super-family, *SrgA*, *SrgB*, *SrgC*, *SrgD*, and *SrgE*, and one member of the ARF/SAR subfamily, *SarA *[31,34]. Our *A. niger *sequences included the earlier identified *SarA *(Asp4377), *SrgA *(Asp5114, Asp4222), *SrgB *(Asp3374, Asp70) and *SrgE *(Asp1610) genes. We also identified contigs Asp1708, which encodes a protein with 47% similarity to the *S. cerevisiae *GTP-binding protein YPT52 [35], and Asp1824 and Asp1217 that code for proteins with 87% and 94% identity with *Aspergillus nidulans *members of the Rab subfamily of small GTPases [36].

Post-translational modifications such as glycosylations are often important for the production of biologically active secreted proteins. For instance, introducing an N-glycosylation site into bovine chymosin increased the amount of secreted chymosin expressed by *A. niger *10-fold [37]. Identification of the various genes involved in *O*- and *N*- linked glycosylations [38] would facilitate efforts to engineer the *A. niger *glycosylation pathway. We identified several putative members of the *N*- and *O*-linked protein glycosylation pathways, including; six PTM related O-mannosyltransferases, contigs Asp370, Asp4472, Asp170, Asp1044, Asp1344, and Asp3205 [39,40] and genes that are involved in *N*-linked protein glycosylation such as two contigs, Asp1340, and Asp458, that encode homologs of oligosaccharyl transferases [41].

## Conclusion

The 12,820 ESTs identified in this study represent a major attempt to define the *A. niger *gene set and represent about 5,108 genes. These data dramatically increase the number of identified *A. niger *genes. We have established a searchable web-based database that includes annotations for each EST and the derived contig assemblies to facilitate research community access to this important resource.

Annotation of the phrap unisequences revealed that 83% had a putative homolog in other species, and therefore about 17% represented novel genes. The template cDNA clones, and their derived EST and contig sequences provide a basis for studying the function of individual genes as well as genome-wide studies of the regulatory networks and cellular functions that define *A. niger*. They will also assist gene identification, mapping and annotation efforts once the draft genome sequence of *A. niger *is completed and released. *A. niger*, known for its efficient secretion machinery, is widely used as a host for the production of native and foreign secreted proteins. However, for many proteins problems have arisen in obtaining high amounts in the culture medium. This study identified 399 putative secreted proteins, and 118 proteins that are putatively involved in various steps of the protein secretion pathway. These sequences should facilitate future efforts to engineering *A. niger *strains with improved secretion capabilities for proteins presently difficult to express. Additional details about this study and access to the *A. niger *EST database can be found on our fungal genomics web site [14].

## Methods

### Source material, total and poly (A)^+^RNA isolation

*Aspergillus niger *strain N402, FGSC #4732 was grown at 30°C in Minimal Medium [42] containing 1% w/v of various carbon sources with shaking at 150 RPM. The carbon sources used were: glucose, bran, maltose, xylan, xylose, sorbitol, and lactose. Mycelial samples harvested by filtration and pressed between layers of filter paper to remove excess liquid, were stored at -80°C.

Total RNA was extracted from each mycelial sample. For this, 1.5 g of each frozen mycelial sample was ground to a fine powder in liquid nitrogen. Total RNA was extracted from the powdered mycelial masses using TRIzol^® ^reagent following the manufacturer's recommendations (Invitrogen, Burlington, ON). Total RNA (200 μg) from each culture condition was pooled and the poly(A)^+ ^RNA was purified using oligo-dT cellulose column chromatography (Amersham Biosciences Corp, Piscataway, NJ). Quality and quantification of the RNA were analyzed by running the RNA samples on an Agilent 2100 bioanalyzer (Agilent Technologies, Palo Alto, CA).

### cDNA library construction

The cDNA library was constructed using a Zap-cDNA^® ^Synthesis Kit according to the manufacturer's instructions (Stratagene, La Jolla, CA). Double-stranded cDNA was directionally cloned into the pBluescript^® ^KS ^+ ^vector (Stratagene, La Jolla, CA) between its *Eco*RI (5'-end) and *Xho*I (3'-end) sites and transformed into *E. coli *strain DH5α.

### Plasmid DNA extraction and sequencing

The cDNA library was plated onto LB-ampicillin agar containing X-GAL and IPTG. White colonies were picked and inoculated into 384-well plates containing LB-ampicillin medium using a VersArray robotic colony picker and arrayer system (Bio-Rad, Laboratories, Canada), grown overnight and stored at -70°C after the addition of glycerol (10% v/v). To prepare plasmid DNA from each sample, bacterial inoculates were transferred from the 384 well storage plates to 96-well growth blocks containing 1 ml of 2YT-ampicillin medium per well (Corning, Acton, MA) and grown overnight. Recombinant plasmids were extracted using alkaline lysis [43] and subjected to single-pass sequencing from the T7 universal primer site (5'-end) using an ABI 3730 XL automated sequencing machine (Applied Biosystems, Foster City, CA) at the Génome Québec Innovation Centre (Montreal, PQ).

### Virtual normalization, direct subtraction and selection of colonies forsequencing

Two methods were used to normalize the library. For virtual normalization [16], bacterial colonies harboring independent cDNA clones were arrayed from the 384-well plates onto nitrocellulose membranes, 9,216 colonies per 492-cm^2 ^membrane. The membranes were probed using radiolabeled cDNA. The probe was prepared as follows; double-stranded cDNA was produced from the same mRNA population that was used for library construction using the SMART cDNA construction kit (BD Biosciences, Mississauga, ON) according to the manufacturer's instructions. The double-stranded cDNA was labeled with [^32^P]dCTP by random priming, using the Rediprime™ II Random Prime Labeling System (Amersham Biosciences Corp, Piscataway, NJ). The labeled cDNA was used to probe six membranes, arrayed with 55,296 clones, and the clones were ranked according to the relative intensity of their hybridization signals (Figure 1A). Based on these intensity ratios the colonies were divided into three groups, high (relative intensity 50%-100%), moderate (relative intensity 10%-50%), and weak (relative intensity less than 10%).

For direct subtraction, plasmid DNAs representing each of the non-redundant genes that had already been identified was pooled. The pooled plasmid DNAs were linearized with the restriction endonuclease *Xho*I and radiolabeled "run-off" transcripts were generated using the Riboprobe *in vitro *Transcription System (Promega, Madison, WI). The probe RNA was then used to hybridize to the same membranes that had been subjected to virtual subtraction.

After hybridization, the membranes were exposed to X-ray film, and the intensity of the signal for each colony was quantified using GeneTools image software (Synoptics Limited). The intensity data for each clone was stored in our in-house database. The clones chosen for sequencing were based on the relative intensity of their hybridization signals, determined as a ratio of signal intensity of the individual clone to the maximum signal intensity present on the array.

### Sequence quality control, contig assembly, and sequence analysis

The chromatograms obtained following single pass sequencing of the cDNA clones were processed using three software tools, phred to assign sequence quality values [44,45], lucy to remove vector sequences and regions of low quality sequence [46], and phrap to assemble overlapping sequences into contigs [19]. Sequence similarity searches against the NCBI non-redundant database were conducted using BLASTX [47] with default BLAST parameters. The top 5 scoring BLASTX hits with E values less than e^-5 ^were used to annotate each EST and EST-derived assembly using our annotation program TargetIdentifier [48]. Sequences that did not return alignments with E values less than e^-5 ^were then used to perform BLASTN searches against the NCBI non-redundant nucleotide database. The top 5 BLASTN hits for each query, where the *E *value was required to be < e^-5^, were then used for annotation. The resulting output files are uploaded to a local MySQL database.

Redundancy was also analyzed by means of clustering based on the BLASTN alignments. Sequences that exhibited more than 93% identity over lengths of at least 100 bases were assigned to the same cluster. Cluster assignments were confirmed by additional analysis using ClustalW [49].

For comparing E values obtained by searching databases of different sizes, we normalized the E-values using the following formula:

E_n _= E specific *S nr/S specific,

E_n_: the normalized E value, it is the subject/query E value that would have been obtained had the alignment been generated by searching a database having the same number of amino acids as the NCBI-nr database; E specific: E-value retuned by BLASTX when searching a user specified database other than the NCBI-nr database; S specific: number of amino acids in the user defined database; S nr: number of amino acids in the NCBI-nr database (total 617,284,665).

TargetIdentifier was used to estimate the proportion of the clones that contained complete coding sequences. The criteria used for establishing that a cDNA included the complete ORF can be found on our web site [50].

### Annotation and functional binning

Annotation and functional binning were accomplished using tools provided by the Gene Ontology Consortium [51]. Annotations were based on the Gene Ontology (GO) terms and hierarchical structure [52]. Reference sequences were selected from the BLASTX results with E values less than e-5 obtained by searching the Swiss-Prot database of manually annotated proteins and the TrEMBL database of proteins with automated annotations. The GO categories associated with the BLASTX subject giving the highest score from the Swiss-Prot and TrEMBL databases were used to annotate our *A. niger *singletons and contigs. The GO term annotations were merged and loaded into the AmiGO browser and database [53]. The resulting GO-derived annotations can be viewed with the AmiGO browser at our website [54].

### Signal peptide prediction

The coding region of each singleton and contig was predicted and translated into protein sequences using our OrfPredictor program [55]. The N-terminal 50 amino acids of each predicted polypeptide were searched for a signal peptide using SignalP version 3 [29].

## Authors' contributions

NS normalized the library, prepared the manuscript, and contributed to the gene ontology classification. RS, GB and AT designed the project and the databases, and contributed to the preparation of manuscript. TJ contributed to the culture of the fungus and construction of the library. PG prepared the cDNA for library construction and contributed to the classification of gene ontology terms. PU tracked the clones at different stages of manipulations. XJM contributed to the annotation of the sequences. JS analyzed the raw sequences and contributed to the construction of the EST database.

## Supplementary Material

Additional File 1 ***A. niger *clusters derived from alternatively spliced unisequences**. Table presenting the 56 manually verified clusters that were generated by alternative splicing of 117 phrap unisequences. For each cluster the table includes; the unisequences present in each cluster, the function as assigned by BLAST-based similarity, the BLAST subject species, the GenBank ID for the BLAST subject used for functional assignment, and the Expect value obtained with each unisequence. Click here for file

Additional File 2 **Gene Ontology annotations of the *A. niger *proteins**. Tables presenting the distribution of Gene Ontology classifiers for the 2,549 *A. niger *unisequences that encoded proteins with similarity to protein entries in the GO annotated Swiss-Prot and TrEMBL database. Table A, presents the distribution of 1,696 *A. niger *proteins that could be assigned Biological Process category and subcategory classifiers. Table B, presents the distribution of the 1,195 *A. niger *proteins that could be assigned Cellular Component category and subcategory classifiers. Table C, presents the distribution of the 1,691 *A. niger *proteins that could be assigned Cellular Component category and subcategory classifiers. Click here for file

Additional File 3 ***A. niger *unisequences coding for proteins with a predicted signal peptide**. This file is a table listing the 399 *A. niger *unisequences that code for proteins with a predicted signal peptide. For each unisequence the table includes the unisequence identifier (column indicated Contig), the Mean Value in the output of SiganlP, the position of the signal peptide relative to the predicted N-terminal methionine, the GenBank definition line for the BLAST subject with the lowest Expect value and an assigned function, the GenBank ID for the BLAST subject used as the source of the definition line and the BLAST E value. Click here for file

Additional File 4 **Putative secretory pathway proteins**. This file is a table listing the *A. niger *unisequences that code for proteins that are predicted to function in the secretory pathway. For each unisequence the table includes the unisequence identifier (column designated "Contig"), the predicted function assigned as described for Additional file 3, the GenBank ID for the BLAST subject that provided the predicted function, the BLAST subject organism, the associated Expect value and BLAST score, and the number of identical residues over the number of amino acids in the alignment. Click here for file

## Figures and Tables

**Table 1:** *A. niger *EST summary

Total templates processed	15,052
Total EST sequences obtained	12,820
Average insert size (bp)	1,470
Average length of high quality sequence per EST sequence obtained (bp)	551
Average contig size (bp)	693
Number of clusters	5,108
Number of clones with full-length inserts	2,407
Number of coding sequences completely sequenced	650
Number of clusters derived from more that one unique singleton and/or contig	74
GC content	53.5 %
Full-length ORFs with a potential signal peptide	292
Unknown sequences which encode a signal peptide	107

**Table 2:** Distribution of homology between the unique set of *A. niger *singleton and contig sequences and various databases as determined by BLASTX

	**Number of genes with similarity** ** (E ≤ e**^**-5**^**)**	**Highly significant homology** ** (E ≤ e**^**-30 **^**)**	**Moderate homology** ** (e**^**-30**^** < E ≤ e**^**-10**^**)**	**Weak homology** ** (e**^**-10**^**< E ≤ e**^**-5**^**)**	**Insignificant similarity** ** (e**^**-5**^**< E)**
Total GenBank Database set	4321 (83.06%)	3367 (64.73 %)	811 (15.59%)	143 (2.75%)	881 (16.93%)
Set of predicted proteins for *A. nidulans*	4195 (80.64%)	3313 (63.69%)	682 (13.11%)	200 (3.84%)	1007 (19.36%)
Set of predicted proteins for *N. crassa*	3615 (69.49%)	2261 (43.46%)	995 (19.13%)	359 (6.90%)	1587 (30.51%)
Set of predicted proteins for *P. chrysosporium*	2581 (49.62%)	1292 (24.84%)	864 (16.60%)	425 (8.17%)	2621 (50.38%)
Set of predicted proteins for *S. cerevisiae*	2386 (45.87%)	1110 (21.34%)	803 (15.44%)	359 (6.90%)	2816 (54.13%)

**Table 3:** Comparison of GO profiling among different fungal species

		**% Representation to total in main category**						
		
**Gene Ontology**	**Categories and subcategories**	***S. cerevisiae***	***S. pombe***	***N. crassa***	***P. chrysos-porium***	***A. niger***	***A. nidulans***	***M. grisea***
**Biological process**	metabolism	71.4%	72.7%	76.0%	74.6%	75.6%	71.4%	75.3%
	cellular physiological process	50.3%	46.7%	34.2%	32.6%	30.2%	34.8%	36.9%
	cell growth and/or maintenance	48.5%	45.2%	31.9%	31.6%	29.4%	33.8%	36.2%
	cell communication	4.8%	11.05%	12.8%	4.7%	3.8%	3.9%	4.3%

**Cellular component**	cell	99.3%	99.74%	96.8%	95.5%	98.1%	97.8%	96.5%
	unlocalized	1.0%	0.26%	1.9%	2.7%	0.8%	0.9%	0.8%
	extracellular	0.3%	0.0%	0.7%	2.2%	1.3%	1.3%	2.3%

**Molecular function**	catalytic activity	52.1%	76.46%	54.9%	65.4%	62.6%	60.4%	59.9%
	binding	46.6%	19.47%	45.7%	43.0%	41.8%	41.5%	49.5%
	transporter activity	14.8%	1.50%	11.0%	10.6%	15.5%	12.2%	10.1%
	transcription regulator activity	6.6%	4.28%	4.9%	3.3%	4.6%	6.6%	4.1%
	structural molecule activity	5.3%	0.0%	2.9%	1.9%	2.1%	1.7%	2.2%
	enzyme regulator activity	2.9%	0.86%	1.3%	1.1%	0.7%	0.7%	0.9%
	chaperone activity	2.2%	2.50%	1.5%	1.1%	1.1%	0.8%	1.0%
	signal transducer activity	1.9%	0.0%	1.8%	1.9%	1.5%	1.1%	1.2%
	translation regulator activity	1.4%	0.0%	1.2%	1.1%	1.8%	0.8%	0.8%
