# Supplementary material for: Generation, annotation, and analysis of an extensive Aspergillus niger EST collection
Source: BMC Microbiol. 2006 Feb 2;6:7. doi: 10.1186/1471-2180-6-7 (PMC1434744; doi:10.1186/1471-2180-6-7)
Supplement: Additional File 1 — A. niger clusters derived from alternatively spliced unisequences. Table presenting the 56 manually verified clusters that were generated by alternative splicing of 117 phrap unisequences. For each cluster the table includes; the unisequences present in each cluster, the function as assigned by BLAST-based similarity, the BLAST subject species, the GenBank ID for the BLAST subject used for functional assignment, and the Expect value obtained with each unisequence. [file 1471-2180-6-7-S1.pdf]

Additional file 1 A. niger clusters derived from alternatively spliced unisequences

Cluster	Contigs	Similarity	Organism	ID	E value
1	Asp1<br>Asp48	Similar to mRNA splicing factor srp1	*Schizosaccharomyces pombe*	T48696	1E-23<br>6E-14
2	Asp2<br>Asp36	Similar to hypothetical protein AN4280.2	*Aspergillus nidulans*	EAA58818	1E-06<br>2E-47
3	Asp3<br>Asp279	hypothetical protein AN8791.2	*Aspergillus nidulans*	EAA60584	1E-80<br>1E-106
4	Asp4<br>Asp2183	glucoamylase	*Aspergillus niger*	AAP04499	2E-27<br>0
5	Asp5<br>Asp574<br>Asp25	Similar to hypothetical protein AN2767.2	*Aspergillus nidulans*	EAA63201	1e-76<br>1E-46<br>4E-24
6	Asp6<br>Asp22<br>Asp1810	hypothetical protein AN2289.2d	*Aspergillus nidulans*	EAA64400	nf<br>nf<br>1E-144
7	Asp7<br>Asp55	Homologous to suaprga1	*Emericella nidulans*	CAB62571	8e-80<br>5E-57
8	Asp8<br>Asp50	hypothetical protein AN0856.2	*Aspergillus nidulans*	EAA65686	8E-69<br>5E-23
9	Asp9<br>Asp77	Similar to cutinase transcription factor 1 beta	*Haematonectria haematococca*	P52959	1E-12<br>9E-17
10	Asp10<br>Asp65	Weakly similar to hypothetical protein an6091.2	*Aspergillus nidulans*	EAA58066	6E-23<br>1E-33
11	Asp11<br>Asp51	Similar to hypothetical protein an0129.2	*Aspergillus nidulans*	EAA65307	4e-20<br>nf
12	Asp12<br>Asp1754	hypothetical protein AN3371.2	*Aspergillus nidulans*	EAA63339	nf<br>1E-87
13	Asp13<br>Asp75	Homologous to chaperonin cct4	*Neurospora crassa*	T49506	2E-95<br>4E-94
14	Asp14<br>Asp63	hypothetical protein AN2464.2	*Aspergillus nidulans*	EAA64170	3E-96<br>3E-24
15	Asp15<br>Asp1382	Homologous to benzoylformate decarboxylase	*Neurospora crassa*	CAD70458	2E-88<br>1E-110
16	Asp16<br>Asp96	Similar to peroxisomal membrane protein per10	*Neurospora crassa*	T49696	4E-49<br>4E-09
17	Asp17<br>Asp98	hypothetical protein	*Neurospora crassa*	XP_324525	3E-53<br>4E-21
18	Asp18<br>Asp784<br>Asp400	Similar to hypothetical protein an7686.2	*Aspergillus nidulans*	EAA61872	5E-12<br>9E-33<br>2E-39
19	Asp19<br>Asp875	Weakly similar to hypothetical protein	*Neurospora crassa*	XP_324007	7E-14<br>3E-47
20	Asp20<br>Asp416	UV-endonuclease	*Neurospora crassa*	S55262	5E-12<br>2E-68
21	Asp21<br>Asp47	conserved hypothetical protein	*Aspergillus nidulans*	EAA66134	2E-81<br>3E-65

22	Asp23<br>Asp92	Not found			nf<br>nf
23	Asp24<br>Asp72	hypothetical protein AN5146.2	*Aspergillus nidulans*	EAA62327	8E-78<br>1E-78
24	Asp26<br>Asp1485	hypothetical protein AN6169.2	*Aspergillus nidulans*	EAA57955	1E-69<br>1E-150
25	Asp27<br>Asp1402	Weakly similar to predicted protein	*Neurospora crassa*	XP_325927	nf<br>2E-07
26	Asp28<br>Asp270	hypothetical protein AN8098.2	*Aspergillus nidulans*	EAA59720	nf<br>4E-09
27	Asp29<br>Asp67	Similar to udp-glucose:sterol glycosyltransferase	*Leptosphaeria maculans*	AAM81359	2E-26<br>3E-30
28	Asp30<br>Asp56	phosphatidylserine synthase	*Triticum aestivum*	AAD10497	3E-59<br>4E-35
29	Asp31<br>Asp1160	Similar to hypothetical protein an9515.2	*Aspergillus nidulans*	EAA66749	4E-25<br>5e-37
30	Asp32<br>Asp100	Not found			nf<br>nf
31	Asp33<br>Asp93	hypothetical protein AN4292.2	*Aspergillus nidulans*	EAA60044	6E-29<br>5E-09
32	Asp34<br>Asp82	hypothetical protein AN5226.2	*Aspergillus nidulans*	EAA62407	4E-69<br>1E-26
33	Asp35<br>Asp1454	putative mitochondrial phosphate carrier protein	*Tuber magnatum*	CAB55764	3E-59<br>1E-49
34	Asp43<br>Asp1121	hypothetical protein AN4476.2	*Aspergillus nidulans*	EAA60819	3E-42<br>3E-45
35	Asp45<br>Asp1725	Similar to yeast cortical cytoskeleton component; mammalian cofilin homolog; Cof1p	*Saccharomyces cerevisiae*	NP_013050	2E-19<br>1E-23
36	Asp59<br>Asp2136	Homologous to mitochondrial processing peptidase beta subunit, mitochondrial PRECURSOR	*Neurospora crassa*	XP_331748	1E-69<br>0
37	Asp61<br>Asp2075	transcription factor HACA	*Aspergillus niger*	AAQ73495	5E-93<br>6E-97
38	Asp76<br>Asp2098	Alternative oxidase, mitochondrial precursor	*Aspergillus niger*	O74180	1E-115<br>0
39	Asp79<br>Asp1663	Similar to pali	*Emericella nidulans*	CAA07588	3E-36<br>3E-59
40	Asp83<br>Asp2043	histone h2b	*Emericella nidulans*	P23754	nf<br>3E-44
41	Asp91<br>Asp1621	Similar to hypothetical protein an4699.2	*Aspergillus nidulans*	EAA60741	2E-14<br>1E-125
42	Asp97<br>Asp964	hypothetical protein AN8880.2	*Aspergillus nidulans*	EAA64094	3E-67<br>1E-100
43	Asp295<br>Asp1632	Similar to endosomal protein that regulates cell polarity; similar to ynr048wp and Lem3p; Cdc50p	*Saccharomyces cerevisiae*	NP_010018	1E-71<br>1E-49
44	Asp488<br>Asp1972	hypothetical protein AN1274.2	*Aspergillus nidulans*	EAA65867	9E-59<br>1E-155

45	Asp525<br>Asp1679	hypothetical protein AN0446.2	*Aspergillus nidulans*	EAA66545	3E-48<br>0
46	Asp646<br>Asp1040<br>Asp1976	hypothetical protein AN5840.2	*Aspergillus nidulans*	EAA58349	1E-11<br>1E-11<br>3E-73
47	Asp680<br>Asp1473	hypothetical protein AN3310.2	*Aspergillus nidulans*	EAA63278	3E-40<br>4E-40
48	Asp769<br>Asp1872	hypothetical protein AN2992.2	*Aspergillus nidulans*	EAA63563	4E-18<br>1E-114
49	Asp813<br>Asp1021	hypothetical protein AN3973.2	*Aspergillus nidulans*	EAA59444	1E-41<br>2E-93
50	Asp1046<br>Asp2150	putative thiazole synthase	*Emericella nidulans*	BAD04053	4E-82<br>1E-171
51	Asp1089<br>Asp1919	Similar to serine palmitoyltransferase 2, putative	*Aspergillus fumigatus*	CAE47913	7E-71<br>0
52	Asp1336<br>Asp1749	Subunit IV of cytochrome c oxidase, which is the terminal member of the mitochondrial inner membrane electron transport chain; N-terminal 25 residues of precursor are cleaved during mitochondrial import; Cox4p	*Saccharomyces cerevisiae*	NP_011328	4E-25<br>3E-29
53	Asp1371<br>Asp2170<br>Asp2165	putative Woronin body protein AoHex1	*Aspergillus oryzae*	BAD20636	9E-34<br>2E-64<br>7E-13
54	Asp1617<br>Asp2140	Weakly similar to antifungal protein precursor	*Penicillium chrysogenum*	JC4564	1E-08<br>2E-13
55	Asp1661<br>Asp1860	Not found			nf<br>nf
56	Asp1820<br>Asp2161	Similar to hypothetical protein an8483.2	*Aspergillus nidulans*	EAA67105	1E-09<br>2E-15

nf- no significant homology was found
