# Supplementary material for: Generation, annotation, and analysis of an extensive Aspergillus niger EST collection
Source: BMC Microbiol. 2006 Feb 2;6:7. doi: 10.1186/1471-2180-6-7 (PMC1434744; doi:10.1186/1471-2180-6-7)
Supplement: Additional File 2 — Gene Ontology annotations of the A. niger proteins. Tables presenting the distribution of Gene Ontology classifiers for the 2,549 A. niger unisequences that encoded proteins with similarity to protein entries in the GO annotated Swiss-Prot and TrEMBL database. Table A, presents the distribution of 1,696 A. niger proteins that could be assigned Biological Process category and subcategory classifiers. Table B, presents the distribution of the 1,195 A. niger proteins that could be assigned Cellular Component category and subcategory classifiers. Table C, presents the distribution of the 1,691 A. niger proteins that could be assigned Cellular Component category and subcategory classifiers. [file 1471-2180-6-7-S2.pdf]

Additional file 2 Gene Ontology annotations of the *A. niger* proteins

**Gene Ontology annotation**		**2549**	**100%**			
(a) Biological process		1696	66.54%			
Categories and subcategories		Number of genes	% to total in group			
Metabolism		1282	75.59%			
	protein metabolism	441	34.40%			
				protein biosynthesis	192	43.54%
				protein modification	116	26.30%
				protein catabolism	90	20.41%
				protein folding	28	6.35%
				protein targeting	23	5.22%
				glycoprotein metabolism	13	2.95%
				protein complex assembly	8	1.81%
				other	5	1.13%
	biosynthesis	365	28.47%			
				macromolecule biosynthesis	273	74.79%
				protein biosynthesis	192	54.70%
				amine biosynthesis	52	14.25%
				coenzyme and prosthetic group biosynthesis	28	7.67%
				vitamin biosynthesis	15	4.11%
				aromatic compound biosynthesis	9	2.47%
				organic acid biosynthesis	8	2.19%
				alcohol biosynthesis	7	1.92%
				antibiotic biosynthesis	5	1.37%
				other	16	4.40%
	nucleotide and nucleic acid metabolism	330	25.74%			
				transcription	156	47.27%
				RNA metabolism	72	21.82%
				DNA metabolism	67	20.30%
				nucleotide metabolism	35	10.61%
				nucleobase metabolism	6	1.82%
				gene silencing	4	1.21%
				nucleoside metabolism	4	1.21%
				other	8	1.60%
	carbohydrate metabolism	159	12.40%			
	catabolism	138	10.76%			
				macromolecule catabolism	92	66.67%
				carbohydrate catabolism	29	21.01%
				alcohol catabolism	22	15.94%
				amine catabolism	7	5.07%
				lipid catabolism	6	4.35%
				cell wall catabolism	3	2.17%
				aromatic compound catabolism	2	1.45%
				other	4	2.88%
	organic acid metabolism	136	10.61%			
	electron transport	119	9.28%			
	amine metabolism	114	8.89%			
	amino acid and derivative metabolism	113	8.81%			

	phosphorus metabolism		92	7.18%
	energy pathways		55	4.29%
	lipid metabolism		50	3.90%
	alcohol metabolism		47	3.67%
	coenzyme and prosthetic group		38	2.96%
	aromatic compound metabolism		34	2.65%
	nitrogen metabolism		25	1.95%
	oxidative phosphorylation		21	1.64%
	vitamin metabolism		20	1.56%
	heterocycle metabolism		19	1.48%
	sulfur metabolism		16	1.25%
	oxygen and reactive oxygen species		13	1.01%
	other		30	2.35%
cellular physiological process			512	30.19%
	cell growth and/or maintenance		499	97.46%
		transport	351	70.34%
		cell organization and biogenesis	93	18.64%
		cell proliferation	69	13.83%
		cell homeostasis	11	2.20%
		other	12	2.40%
	cell death		12	2.34%
	sporulation		7	1.37%
	other		5	0.99%
response to stimulus			51	3.00%
secretion			5	0.29%

(b) Cellular component 1195 46.88%

Categories and subcategories				Number of genes	% to total in group
cell				1172	98.08%
	intracellular			787	67.15%
		cytoplasm		473	60.10%
			mitochondrion	103	21.78%
			ribosome	98	20.72%
			endoplasmic reticulum	52	10.99%
			cytoskeleton	45	9.51%
			cytosol	29	6.13%
			Golgi apparatus	19	4.02%
			vacuole	16	3.38%
			cytoplasmic vesicle	15	3.17%
			membrane coat	12	2.54%
			microbody	12	2.54%
			other	22	4.62%
		nucleus		296	37.61%
			nucleolus	18	6.08%
			nucleoplasm	12	4.05%
			proteasome complex	9	3.04%
			nuclear membrane	8	2.70%
			other	4	1.36%
		cell cortex		9	1.14%

	chromosome			9	1.14%
	other			7	0.89%
membrane		483	41.21%		
	integral to membrane			257	53.21%
	inner membrane			34	7.04%
	mitochondrial membrane			34	7.04%
	plasma membrane			26	5.38%
	endomembrane system			19	3.93%
	outer membrane			9	1.86%
	vacuolar membrane			7	1.45%
	other			7	1.45%
extracellular		15	1.26%		
Unlocalized		10	0.84%		

(c) Molecular function 1691 66.34%

Categories and subcategories		Number of genes	% to total in group		
catalytic activity		1059	62.63%		
	hydrolase activity			339	32.01%
	oxidoreductase activity			274	25.87%
	transferase activity			261	24.65%
	kinase activity			115	10.86%
	ligase activity			67	6.33%
	lyase activity			66	6.23%
	helicase activity			36	3.40%
	isomerase activity			29	2.74%
	other			8	1.13
binding		707	41.81%		
	nucleotide binding			319	45.12%
	nucleic acid binding			203	28.71%
	metal ion binding			186	26.31%
	protein binding			32	4.53%
	cofactor binding			28	3.96%
	lipid binding			6	0.85%
	amino acid binding			5	0.71%
	other			11	1.54%
transporter		262	15.49%		
	amine/polyamine transporter			147	56.11%
	carbohydrate transporter			79	30.15%
	carrier			44	16.79%
	channel/pore class transporter			35	13.36%
	drug transporter			25	9.54%
	electron transporter			19	7.25%

	intracellular transporter		19	7.25%
	ion transporter		9	3.44%
	lipid transporter		8	3.05%
	nucleotide and nucleic acid transporter		7	2.67%
	other		12	4.57%
transcription regulator	78	4.61%		
structural molecule	35	2.07%		
translation regulator	30	1.77%		
signal transducer	25	1.48%		
chaperone	18	1.06%		
enzyme regulator	11	0.65%		
antioxidant	8	0.47%		
motor	3	0.18%		
nutrient reservoir	3	0.18%		

(a) 1696 unigenes generated 1850 multiple mappings. Percentage representation is based on 1696. (b) 1195 unigenes generated 1197
multiple mappings. Percentage representation is based on 1195. (c) 1691 unigenes generated 2239 multiple mappings. Percentage representation is based on 1691.
