# Supplementary material for: Generation, annotation, and analysis of an extensive Aspergillus niger EST collection
Source: BMC Microbiol. 2006 Feb 2;6:7. doi: 10.1186/1471-2180-6-7 (PMC1434744; doi:10.1186/1471-2180-6-7)
Supplement: Additional File 3 — A. niger unisequences coding for proteins with a predicted signal peptide. This file is a table listing the 399 A. niger unisequences that code for proteins with a predicted signal peptide. For each unisequence the table includes the unisequence identifier (column indicated Contig), the Mean Value in the output of SiganlP, the position of the signal peptide relative to the predicted N-terminal methionine, the GenBank definition line for the BLAST subject with the lowest Expect value and an assigned function, the GenBank ID for the BLAST subject used as the source of the definition line and the BLAST E value. [file 1471-2180-6-7-S3.pdf]

Additional file 3 *A. niger* unisequences coding for proteins with a predicted signal peptide

Contig	Mean Value	Position of Signal peptide	Similarity	ID	Organism	E value
Asp1876	0.895	1-21(21)	1,4-beta-d-glucan cellobiohydrolase b precursor	AAF04492	Aspergillus niger	1.00E-112
Asp2002	0.889	1-23(23)	acetyl xylan esterase	AAK60128	Aspergillus ficuum	1.00E-171
Asp1080	0.797	1-20(20)	acid phosphatase precursor (ph 6-optimum acid phosphatase) (apase6)	Q12546	Aspergillus ficuum	0
Asp2114	0.896	1-24(24)	alpha-amylase	BAD06003	Aspergillus awamori	0
Asp4255	0.973	1-17(17)	alpha-amylase amya	AAF17103	Emericella nidulans	2.00E-21
Asp385	0.947	1-16(16)	alpha-galactosidase	CAB46229	Aspergillus niger	1.00E-151
Asp2882	0.556	1-21(21)	alpha-galactosidase c precursor (melibiase)	Q9UUZ4	Aspergillus niger	1.00E-145
Asp2103	0.908	1-19(19)	alpha-glucosidase precursor (maltase)	P56526	Aspergillus niger	0
Asp1584	0.955	1-20(20)	alpha-glucuronidase	CAC38119	Aspergillus niger	1.00E-134
Asp5072	0.787	1-18(18)	alpha-l-arabinofuranosidase b precursor (arabinosidase b)	P42255	Aspergillus niger	1.00E-105
Asp2120	0.645	1-26(26)	alpha-l-arabinofuranosidase precursor (arabinoxylan arabinofuranohydrolase)	P79019	Aspergillus niger	0
Asp1666	0.886	1-18(18)	aspergillopepsin ii precursor (acid protease a) (proctase a)	P24665	Aspergillus niger	1.00E-133
Asp633	0.784	1-25(25)	ataap	AAD37345	Emericella nidulans	6.00E-87
Asp1330	0.826	1-18(18)	carboxypeptidase cpds precursor	P52719	Aspergillus saitoi	1.00E-132
Asp1439	0.693	1-15(15)	catalase	BAC55897	Aspergillus oryzae	0
Asp2003	0.776	1-16(16)	catalase r	P55303	Aspergillus niger	0
Asp1784	0.85	1-21(21)	cinnamoyl esterase faea	AAK60631	Aspergillus niger	1.00E-164
Asp2081	0.914	1-25(25)	cytochrome p450 sterol 14 alpha-demethylase	AAF32372	Aspergillus fumigatus	1.00E-134
Asp1020	0.898	1-19(19)	disulfide isomerase tiga precursor	Q00216	Aspergillus niger	0
Asp2160	0.845	1-19(19)	endo-1,4-beta-xylanase a precursor (xylanase a) (1,4-beta-d-xylan xylanohydrolase A)	P33559	Aspergillus kawachii	1.00E-142

Asp2049	0.834	1-18(18)	endo-1,4-beta-xylanase ii precursor (xylanase ii) (1,4-beta-d-xylan xylanohydrolase II)	P55330	Aspergillus niger	1.00E-121
Asp1819	0.763	1-16(16)	endoglucanase a	CAA11964	Aspergillus niger	1.00E-138
Asp1967	0.81	1-18(18)	endoglucanase b	CAA11965	Aspergillus niger	1.00E-162
Asp3520	0.915	1-21(21)	endosomal p24b protein precursor, putative	EAA66272	Aspergillus fumigatus	3.00E-99
Asp2183	0.893	1-18(18)	glucoamylase g1 and g2 precursor (glucan 1,4-alpha-glucosidase) (1,4-alpha-D-glucan glucohydrolase)	P04064	Aspergillus niger	0
Asp158	0.754	1-34(34)	Homologous to aar149wp	NP_982692	Eremothecium gossypii	5.00E-47
Asp1652	0.816	1-27(27)	Homologous to abr129cp	NP_983076	Eremothecium gossypii	1.00E-42
Asp962	0.957	1-24(24)	hypothetical protein AN0871.2	EAA65900	Aspergillus nidulans	2.00E-78
Asp3863	0.586	1-22(22)	hypothetical protein AN4730.2	EAA60772	Aspergillus nidulans	5.00E-44
Asp736	0.932	1-19(19)	Homologous to allergen asp f 15 precursor (asp f 13)	O60022	Aspergillus fumigatus	8.00E-31
Asp4287	0.7	1-18(18)	Homologous to allergen asp f 4	O60024	Aspergillus fumigatus	2.00E-09
Asp1521	0.884	1-21(21)	Homologous to aspartic protease	BAC00848	Aspergillus oryzae	4.00E-81
Asp1268	0.912	1-18(18)	Homologous to aspergillopepsin ii precursor (acid protease a) (proctase a)	P24665	Aspergillus niger	4.00E-78
Asp1143	0.655	1-21(21)	Homologous to beta (1-3) glucanosyltransferase gel2p [aspergillus fumigatus]	AAF40139	Aspergillus fumigatus	1.00E-112
Asp1624	0.837	1-23(23)	Homologous to c-8 sterol isomerase erg-1	CAC28749	Neurospora crassa	1.00E-89
Asp1888	0.817	1-18(18)	Homologous to cellobiohydrolase ii	AAL33604	Talaromyces emersonii	1.00E-120
Asp2026	0.828	1-21(21)	Homologous to cellobiohydrolase ii	AAL33604	Talaromyces emersonii	1.00E-145
Asp1377	0.737	1-26(26)	Homologous to chain a, crystal structure of oxalate decarboxylase	1J58	Bacillus subtilis	1.00E-61
Asp3187	0.505	1-20(20)	Homologous to cis-prenyltransferase	CAD21109	Neurospora crassa	2.00E-17
Asp2179	0.821	1-20(20)	Homologous to epd1 protein precursor (essential for pseudohyphal development 1)	P56092	Candida maltosa	1.00E-157
Asp3286	0.795	1-22(22)	Homologous to exo-1,3-beta-glucanase	Q7Z9L3	Aspergillus oryzae	6.00E-69
Asp1997	0.805	1-19(19)	Homologous to gel1 protein	AAC35942	Aspergillus fumigatus	0
Asp1825	0.656	1-21(21)	Homologous to glucose		Hypocrea jecorina	0

			transporter; trhxt1	AAR23147		
Asp570	0.911	1-19(19)	Homologous to glutaminase a	BAA86935	Emericella nidulans	2.00E-71
Asp1746	0.706	1-24(24)	Homologous to glycerol-3-phosphate dehydrogenase precursor related protein [mips]	XP_325309	Neurospora crassa	0
Asp956	0.68	1-17(17)	Homologous to hypothetical protein an0672.2	EAA65448	Aspergillus nidulans	1.00E-109
Asp1875	0.901	1-23(23)	Homologous to hypothetical protein an0819.2	EAA65649	Aspergillus nidulans	5.00E-82
Asp3597	0.862	1-21(21)	Homologous to hypothetical protein an0867.2	EAA65896	Aspergillus nidulans	1.00E-94
Asp1823	0.661	1-27(27)	Homologous to hypothetical protein an1465.2	EAA64595	Aspergillus nidulans	1.00E-177
Asp3195	0.875	1-17(17)	Homologous to hypothetical protein an1855.2	EAA65020	Aspergillus nidulans	9.00E-57
Asp1565	0.874	1-21(21)	Homologous to hypothetical protein an1950.2	EAA65115	Aspergillus nidulans	4.00E-76
Asp3634	0.814	1-22(22)	Homologous to hypothetical protein an2056.2	EAA64888	Aspergillus nidulans	9.00E-97
Asp2094	0.885	1-17(17)	Homologous to hypothetical protein an3079.2	EAA63650	Aspergillus nidulans	2.00E-50
Asp1553	0.65	1-23(23)	Homologous to hypothetical protein an3184.2	EAA62948	Aspergillus nidulans	6.00E-83
Asp723	0.56	1-33(33)	Homologous to hypothetical protein an3705.2	EAA59913	Aspergillus nidulans	2.00E-64
Asp4879	0.813	1-21(21)	Homologous to hypothetical protein an4209.2	EAA59308	Aspergillus nidulans	2.00E-64
Asp2723	0.957	1-17(17)	Homologous to hypothetical protein an4257.2	EAA58925	Aspergillus nidulans	1.00E-06
Asp343	0.909	1-19(19)	Homologous to hypothetical protein an4728.2	EAA60770	Aspergillus nidulans	1.00E-108
Asp2362	0.754	1-16(16)	Homologous to hypothetical protein an5050.2	EAA62712	Aspergillus nidulans	3.00E-17
Asp519	0.688	1-24(24)	Homologous to hypothetical protein an5619.2	EAA62712	Aspergillus nidulans	1.00E-73
Asp2880	0.53	1-23(23)	Homologous to hypothetical protein an6149.2	EAA57935	Aspergillus nidulans	4.00E-55
Asp1693	0.506	1-43(43)	Homologous to hypothetical protein an6689.2	EAA57632	Aspergillus nidulans	1.00E-104
Asp911	0.855	1-19(19)	Homologous to hypothetical protein an7035.2	EAA61681	Aspergillus nidulans	3.00E-24
Asp1998	0.949	1-16(16)	Homologous to hypothetical protein an7102.2	EAA61307	Aspergillus nidulans	2.00E-51
Asp5201	0.84	1-17(17)	Homologous to hypothetical protein an7168.2	EAA61420	Aspergillus nidulans	4.00E-68
Asp2110	0.753	1-22(22)	Homologous to hypothetical protein an7735.2	EAA61250	Aspergillus nidulans	2.00E-50
Asp2113	0.78	1-22(22)	Homologous to hypothetical protein an7950.2	EAA59604	Aspergillus nidulans	1.00E-118

Asp3715	0.961	1-20(20)	Homologous to hypothetical protein an8194.2	EAA58838	Aspergillus nidulans	3.00E-14
Asp3624	0.541	1-25(25)	Homologous to hypothetical protein an8421.2	EAA67043	Aspergillus nidulans	2.00E-60
Asp2735	0.559	1-24(24)	Homologous to hypothetical protein an8467.2	EAA67089	Aspergillus nidulans	4.00E-46
Asp4529	0.583	1-35(35)	Homologous to hypothetical protein an8700.2	EAA60249	Aspergillus nidulans	1.00E-50
Asp2557	0.619	1-37(37)	Homologous to hypothetical protein fg01043.	XP_381219	Gibberella zeae	1.00E-43
Asp1756	0.735	1-21(21)	Homologous to lysophospholipase	AAQ85122	Aspergillus fumigatus	0
Asp1987	0.619	1-20(20)	Homologous to mannose-1-phosphate guanylyltransferase	AAC39498	Hypocrea jecorina	1.00E-163
Asp4391	0.703	1-16(16)	Homologous to mitochondrial nicotinamide nucleotide transhydrogenase-related protein [imported]	T48756	Neurospora crassa	3.00E-23
Asp4964	0.677	1-37(37)	Homologous to nadph oxidase	AAN75017	Emericella nidulans	1.00E-63
Asp364	0.801	1-23(23)	Homologous to pectine lyase f	CAD34589	Aspergillus niger	3.00E-45
Asp1885	0.853	1-19(19)	Homologous to pepsin-type protease	AAL40802	Talaromyces emersonii	2.00E-56
Asp1612	0.898	1-20(20)	Homologous to phosphatidylglycerol/phosphatidylinositol transfer protein	AAD16095	Aspergillus oryzae	3.00E-65
Asp4168	0.81	1-19(19)	Homologous to possible secreted cellulose-binding protein	CAF32158	Aspergillus fumigatus	3.00E-59
Asp1090	0.811	1-30(30)	Homologous to predicted protein	EAA61876	Aspergillus nidulans	2.00E-45
Asp4738	0.512	1-24(24)	Homologous to predicted protein	XP_325931	Neurospora crassa	2.00E-22
Asp4603	0.477	1-27(27)	Homologous to proteasome regulatory subunit 12	AAB84057	Hypocrea jecorina	2.00E-55
Asp3309	0.505	1-16(16)	Homologous to pyridoxine	AAK50016	Aspergillus nidulans	3.00E-98
Asp1210	0.538	1-21(21)	Homologous to pyruvate dehydrogenase beta chain precursor (pdb1) [mips]	CAG24029	Aspergillus niger	3.00E-95
Asp1911	0.89	1-19(19)	Homologous to rasp f 9	CAA11266	Aspergillus fumigatus	2.00E-89
Asp2240	0.564	1-20(20)	Homologous to ribosomal protein l15 homologue, putative	CAE47918	Aspergillus fumigatus	6.00E-74
Asp1267	0.705	1-18(18)	Homologous to sun family protein, putative	CAF32103	Aspergillus fumigatus	5.00E-73
Asp1946	0.825	1-21(21)	Homologous to tal1 [saccharomyces kluyveri]	AAO32594	Saccharomyces kluyveri	5.00E-78
Asp2308	0.932	1-22(22)	Homologous to udp-n-acetylglucosamine:dolichyl phosphate N-acetylglucosamine-1-	AAL78196	Aspergillus niger	9.00E-22

			phosphate transferase; GPT			
Asp3401	0.874	1-25(25)	Homologous to unknown	AAC61875	Emericella nidulans	1.00E-110
Asp4894	0.723	1-25(25)	hypothetical protein an1865.2	EAA65030	Aspergillus nidulans	4.00E-74
Asp2091	0.533	1-16(16)	hypothetical protein an3591.2	EAA59799	Aspergillus nidulans	0
Asp1101	0.726	1-19(19)	hypothetical protein an4559.2	EAA60902	Aspergillus nidulans	1.00E-48
Asp1572	0.777	1-33(33)	hypothetical protein an9491.2	EAA66772	Aspergillus nidulans	1.00E-85
Asp1588	0.478	1-32(32)	imilar to hypothetical protein fg05266.1]	XP_385442	Gibberella zeae	3.00E-06
Asp1956	0.511	1-24(24)	ketol-acid reductoisomerase precursor (acetohydroxy-acid REDUCTOISOMERASE) (ALPHA-KETO-BETA-HYDROXYLACIL REDUCTOISOMERASE)	XP_322910	Neurospora crassa	1.00E-136
Asp177	0.766	1-19(19)	kexin precursor	CAB64692	Aspergillus niger	7.00E-80
Asp521	0.798	1-21(21)	mannosyl-oligosaccharide 1,2-alpha-mannosidase (ec 3.2.1.113) precursor	S63701	Aspergillus phoenicis	1.00E-105
Asp1455	0.878	1-36(36)	mipc synthase; sura	AAP47108	Aspergillus nidulans	2.00E-73
Asp1512	0.871	1-21(21)	nadph--cytochrome p450 reductase (cpr) (p450r)	Q00141	Aspergillus niger	1.00E-132
Asp1340	0.835	1-23(23)	oligosaccharyltransferase alpha subunit	AAK08631	Aspergillus niger	1.00E-101
Asp2121	0.751	1-18(18)	pepsinogen	AAA20876	Aspergillus niger	0
Asp1902	0.92	1-20(20)	protein disulfide isomerase precursor (pdi)	Q12730	Aspergillus niger	1.00E-131
Asp4412	0.818	1-23(23)	rhamnogalacturonan acetyl esterase	CAC41360	Aspergillus niger	3.00E-63
Asp149	0.811	1-23(23)	ribonuclease m (rnase m)	P19791	Aspergillus saitoi	1.00E-129
Asp2027	0.844	1-15(15)	serine-type carboxypeptidase homolog precursor	JC7666	Emericella nidulans	0
Asp1212	0.884	1-20(20)	hypothetical protein AN4515.2	EAA60858	Aspergillus nidulans	9.00E-62
Asp3072	0.501	1-20(20)	Similar to 3-oxoacyl-[acyl-carrier-protein]	AAB81078	Neurospora crassa	1.00E-39
Asp4049	0.95	1-21(21)	Similar to aal005wp	NP_982537	Eremothecium gossypii	1.00E-13
Asp1938	0.555	1-20(20)	Similar to acidic ribosomal protein p2	AAG01801	Aspergillus fumigatus	2.00E-20
Asp1450	0.778	1-20(20)	hypothetical protein AN3918.2	EAA59227	Aspergillus nidulans	5.00E-107

Asp603	0.486	1-44(44)	Similar to aer063wp	NP_984923	Eremothecium gossypii	4.00E-10
Asp3968	0.697	1-18(18)	Similar to aer274wp	NP_985131	Eremothecium gossypii	2.00E-14
Asp36	0.508	1-23(23)	hypothetical protein AN4280.2	EAA58818	Aspergillus nidulans	2.00E-47
Asp1321	0.939	1-19(19)	hypothetical protein AN1176.2	EAA66294	Aspergillus nidulans	1.00E-23
Asp2106	0.827	1-21(21)	Similar to allergen asp f 7	O42799	Aspergillus fumigatus	1.00E-15
Asp170	0.88	1-25(25)	Similar to alpha-1,2-mannosyltransferase; ktr3p	NP_009764	Saccharomyces cerevisiae	4.00E-25
Asp1434	0.84	1-25(25)	Similar to alpha-amylase amya [emericella nidulans]	AAF17103	Emericella nidulans	1.00E-100
Asp1263	0.919	1-17(17)	Similar to alpha-galactosidase, putative / melibiase, putative / alpha-D-galactoside galactohydrolase, putative	NP_568193	Arabidopsis thaliana	2.00E-62
Asp2140	0.936	1-18(18)	Similar to antifungal protein precursor	JC4564	Penicillium chrysogenum	2.00E-13
Asp1488	0.595	1-18(18)	Similar to aspartic protease	BAC00848	Aspergillus oryzae	9.00E-56
Asp4230	0.781	1-17(17)	Similar to beta-glucosidase (ec 3.2.1.21) precursor - yeast	JC4376	Candida molischiana	1.00E-16
Asp2128	0.709	1-18(18)	Similar to beta-glucosidase 5 [coccidioides posadasii]	AAL09829	Coccidioides posadasii	1.00E-76
Asp4235	0.795	1-18(18)	Similar to c4-dicarboxylate transport protein mae1	CAD21205	Neurospora crassa	7.00E-32
Asp2166	0.899	1-17(17)	Similar to cell wall protein	BAD01559	Aspergillus kawachii	8.00E-80
Asp925	0.758	1-20(20)	Similar to cellobiose dehydrogenase	AAC26221	Thielavia heterothallica	2.00E-21
Asp422	0.683	1-27(27)	Similar to chaperonin, t-complex-type - fission yeast	T43202	Schizosaccharomyces pombe	1.00E-75
Asp1594	0.889	1-27(27)	Similar to class i alpha-mannosidase 1a	AAG48160	Aspergillus nidulans	1.00E-142
Asp5041	0.89	1-21(21)	Similar to crh-like protein	AAN87849	Aspergillus fumigatus	8.00E-33
Asp2243	0.697	1-27(27)	Similar to ctns protein	CAC28641	Neurospora crassa	8.00E-28
Asp1787	0.797	1-27(27)	Similar to endo-1,4-beta-xylanase b precursor (xylanase b) (1,4-beta-d-xylan xylanohydrolase B)	P48824	Aspergillus kawachii	2.00E-41
Asp2037	0.937	1-15(15)	Similar to endo-alpha-1,5-arabinanase precursor	AF300878	Aspergillus aculeatus	5.00E-97
Asp4470	0.814	1-21(21)	Similar to endoglucanase iv precursor (endo-1,4-beta-glucanase iv) (cellulase IV) (EGIV)	O14405	Hypocrea jecorina	3.00E-20

Asp1498	0.703	1-22(22)	Similar to endoglucanase iv precursor (endo-1,4-beta-glucanase iv) (cellulase IV) (EGIV)	O14405	Hypocrea jecorina	1.00E-64
Asp425	0.602	1-25(25)	Similar to endoglucanase, putative	CAF31975	Aspergillus fumigatus	9.00E-28
Asp1893	0.554	1-19(19)	hypothetical protein AN1543.2	EAA64250	Aspergillus nidulans	0.0
Asp3384	0.82	1-22(22)	Similar to gaba permease	CAB43936	Emericella nidulans	4.00E-16
Asp2363	0.739	1-26(26)	Similar to glutathione-disulfide reductase (ec 1.8.1.7) [similarity]	T51908	Neurospora crassa	7.00E-27
Asp2440	0.938	1-24(24)	Similar to high affinity methionine permease; mup1p	NP_011569	Saccharomyces cerevisiae	1.00E-54
Asp3554	0.813	1-21(21)	hypothetical protein	XP_329264	Neurospora crassa	3.00E-41
Asp3391	0.808	1-32(32)	hypothetical protein AN0315.2	EAA65721	Aspergillus nidulans	7.00E-26
Asp1767	0.616	1-36(36)	hypothetical protein AN1719.2	EAA64005	Aspergillus nidulans	1.00E-52
Asp1575	0.879	1-16(16)	Similar to hypothetical protein an2237.2	EAA63922	Aspergillus nidulans	2.00E-62
Asp63	0.513	1-16(16)	Similar to hypothetical protein an2464.2	EAA64170	Aspergillus nidulans	3.00E-24
Asp1261	0.632	1-20(20)	Similar to hypothetical protein an3046.2	EAA63617	Aspergillus nidulans	5.00E-28
Asp126	0.792	1-33(33)	Similar to hypothetical protein an3057.2	EAA63628	Aspergillus nidulans	7.00E-17
Asp5185	0.734	1-19(19)	Similar to hypothetical protein an3520.2	EAA59081	Aspergillus nidulans	8.00E-50
Asp4639	0.522	1-19(19)	Similar to hypothetical protein an3744.2	EAA59952	Aspergillus nidulans	4.00E-47
Asp1649	0.82	1-19(19)	Similar to hypothetical protein an3925.2	EAA59234	Aspergillus nidulans	6.00E-48
Asp3794	0.929	1-23(23)	Similar to hypothetical protein an4095.2	EAA59356	Aspergillus nidulans	7.00E-42
Asp1032	0.935	1-17(17)	Similar to hypothetical protein an4190.2	EAA59289	Aspergillus nidulans	7.00E-14
Asp4612	0.917	1-29(29)	Similar to hypothetical protein an4895.2	EAA60973	Aspergillus nidulans	1.00E-46
Asp446	0.9	1-26(26)	Similar to hypothetical protein an6371.2	EAA58755	Aspergillus nidulans	2.00E-67
Asp4793	0.919	1-18(18)	Similar to hypothetical protein an6754.2	EAA58572	Aspergillus nidulans	9.00E-28
Asp109	0.819	1-25(25)	Similar to hypothetical protein an6984.2	EAA61630	Aspergillus nidulans	4.00E-46
Asp1995	0.879	1-20(20)	Similar to hypothetical protein an7329.2	EAA61380	Aspergillus nidulans	8.00E-09
Asp4036	0.922	1-26(26)	Similar to hypothetical protein an7754.2	EAA61542	Aspergillus nidulans	2.00E-17

Asp1986	0.85	1-18(18)	Similar to hypothetical protein an7785.2	EAA61573	Aspergillus nidulans	2.00E-49
Asp1134	0.938	1-20(20)	Similar to hypothetical protein an8043.2	EAA59665	Aspergillus nidulans	6.00E-35
Asp2097	0.87	1-24(24)	Similar to hypothetical protein an8063.2	EAA59685	Aspergillus nidulans	1.00E-148
Asp386	0.847	1-20(20)	Similar to hypothetical protein an8175.2	EAA59197	Aspergillus nidulans	5.00E-55
Asp1167	0.773	1-18(18)	Similar to hypothetical protein an8395.2	EAA67017	Aspergillus nidulans	8.00E-48
Asp614	0.732	1-45(45)	Similar to hypothetical protein an9118.2	EAA61951	Aspergillus nidulans	1.00E-07
Asp558	0.931	1-19(19)	Similar to hypothetical protein an9156.2	EAA61989	Aspergillus nidulans	3.00E-41
Asp247	0.921	1-18(18)	Similar to hypothetical protein an9384.	EAA66451	Aspergillus nidulans	2.00E-96
Asp2050	0.932	1-18(18)	Similar to hypothetical protein fg10461.1	XP_390637	Gibberella zeae	1.00E-98
Asp3871	0.487	1-33(33)	Similar to hypothetical protein fg10685.1	XP_390861	Gibberella zeae	3.00E-34
Asp1466	0.901	1-18(18)	Similar to hypothetical protein mg06169.4	EAA53041	Magnaporthe grisea	2.00E-75
Asp510	0.953	1-17(17)	Similar to hypothetical protein mg09179.4 [magnaporthe grisea 70-15]	EAA55372	Magnaporthe grisea	5.00E-47
Asp3126	0.922	1-17(17)	Similar to l-asparaginase 3 precursor (l-asparagine amidohydrolase 3)	Q8NKC0	Schizosaccharomyces pombe	1.00E-58
Asp1870	0.849	1-23(23)	hypothetical protein AN6307.2	EAA58691	Aspergillus nidulans	1.00E-46
Asp1029	0.84	1-19(19)	Similar to lipase precursor	O59952	Triacylglycerol lipase	1.00E-43
Asp1416	0.717	1-19(19)	Similar to lipase precursor	O59952	Triacylglycerol lipase	3.00E-48
Asp3547	0.769	1-16(16)	Similar to lysophospholipase	AAQ85123	Aspergillus fumigatus	4.00E-54
Asp1109	0.863	1-36(36)	Similar to mnn9p	AAK40024	Pichia angusta	1.00E-20
Asp1949	0.93	1-19(19)	Similar to mutanase (muta) gene [imported]	T49781	Neurospora crassa	1.00E-84
Asp3951	0.52	1-21(21)	Similar to nfrl	BAA22375	Xenopus laevis	1.00E-12
Asp79	0.965	1-26(26)	Similar to pali	CAA07588	Emericella nidulans	3.00E-36
Asp1184	0.561	1-18(18)	Similar to pcl-like cyclin pas1	NP_593306	Schizosaccharomyces pombe	1.00E-10
Asp4244	0.63	1-29(29)	hypothetical protein AN4514.2	EAA60857	Aspergillus nidulans	4.00E-49
Asp3885	0.903	1-27(27)	hypothetical protein AN0049	EAA65368	Aspergillus nidulans	2.00E-54
Asp1163	0.776	1-22(22)	Similar to predicted protein		Aspergillus	1.00E-66

				EAA57696	nidulans	
Asp3835	0.583	1-25(25)	hypothetical protein AN5458	EAA62618	Aspergillus nidulans	3.00E-42
Asp4295	0.817	1-49(49)	Similar to predicted protein	XP_325502	Neurospora crassa	1.00E-23
Asp3923	0.857	1-19(19)	Similar to predicted protein	XP_326219	Neurospora crassa	1.00E-42
Asp1392	0.957	1-22(22)	Similar to predicted protein	XP_328587	Neurospora crassa	1.00E-22
Asp1473	0.914	1-18(18)	Similar to predicted protein	XP_323846	Neurospora crassa	6.00E-13
Asp2142	0.843	1-25(25)	Similar to predicted protein	XP_325061	Neurospora crassa	4.00E-55
Asp2168	0.593	1-22(22)	Similar to predicted protein	XP_327422	Neurospora crassa	3.00E-09
Asp294	0.931	1-28(28)	Similar to predicted protein	XP_331987	Neurospora crassa	1.00E-19
Asp650	0.931	1-16(16)	Similar to predicted protein	XP_324909	Neurospora crassa	7.00E-27
Asp680	0.914	1-18(18)	Similar to predicted protein	XP_325522	Neurospora crassa	4.00E-13
Asp780	0.865	1-16(16)	Similar to predicted protein	XP_327076	Neurospora crassa	6.00E-20
Asp811	0.717	1-35(35)	Similar to predicted protein	XP_330405	Neurospora crassa	9.00E-09
Asp4347	0.967	1-20(20)	Similar to protein	CAE76395	Neurospora crassa	9.00E-40
Asp3534	0.817	1-29(29)	Similar to protein that forms a heterotrimeric complex with erp2p, emp24p, and Erv25p; member, along with Emp24p and Erv25p, of the p24 family involved in ER to Golgi transport and localized to COPII-coated vesicles; Erp1p	NP_009402	Saccharomyces cerevisiae	7.00E-41
Asp4108	0.481	1-41(41)	Similar to protein, with a transmembrane domain (3k918) [Caenorhabditis elegans]	NP_499181	Caenorhabditis elegans	5.00E-07
Asp3350	0.869	1-16(16)	hypothetical protein AN4505.2	EAA60848	Aspergillus nidulans	1.00E-08
Asp4546	0.696	1-23(23)	Similar to stcb_emeni probable sterigmatocystin biosynthesis p450 monooxygenase STCB (Cytochrome P450 62)]	Q12608	Aspergillus nidulans	4.00E-15
Asp4899	0.925	1-29(29)	Similar to suppressor of pi four kinase; sfk1p	NP_012873	Saccharomyces cerevisiae	6.00E-07
Asp742	0.85	1-21(21)	Similar to thioredoxin	P29429	Emericella nidulans	9.00E-23
Asp1415	0.855	1-19(19)	Similar to tripeptidylpeptidase 2	CAE17675	Aspergillus fumigatus	3.00E-66
Asp3430	0.901	1-19(19)	hypothetical protein AN2497.2	EAA63982	Aspergillus nidulans	9.00E-49

Asp4598	0.892	1-30(30)	Weakly similar to alpha 1,6 mannosyltransferase	CAD91643	Yarrowia lipolytica	5.00E-08
Asp1295	0.912	1-24(24)	Similar to wsc4 homolog [imported]	T45525	Kluyveromyces lactis	2.00E-17
Asp1086	0.939	1-16(16)	Similar to ycl012cp	AAN86070	Saccharomyces cerevisiae	6.00E-14
Asp1712	0.553	1-46(46)	hypothetical protein AN2299.2	EAA64410	Aspergillus nidulans	0
Asp3507	0.671	1-31(31)	hypothetical protein AN5599.2	EAA62242	Aspergillus nidulans	2.00E-44
Asp2153	0.951	1-16(16)	subtilisin-like serine protease pepc precursor	P33295	Aspergillus niger	0
Asp53	0.859	1-19(19)	sugar transporter	AAL89823	Aspergillus niger	1.00E-116
Asp1821	0.476	1-32(32)	vacuolar membrane atpase c	BAB62811	Aspergillus oryzae	7.00E-73
Asp957	0.858	1-26(26)	hypothetical protein AN7457.2	EAA62037	Aspergillus nidulans	6.00E-50
Asp393	0.761	1-19(19)	Weakly similar to aar128wp]	NP_982670	Eremothecium gossypii	5.00E-34
Asp1345	0.887	1-29(29)	Weakly similar to adl096wp	NP_984000	Eremothecium gossypii	2.00E-46
Asp1201	0.959	1-19(19)	hypothetical protein AN4814.2	EAA60384	Aspergillus nidulans	2.00E-34
Asp4874	0.632	1-34(34)	Weakly similar to aflatoxin biosynthesis ketoreductase nor-1	Q00278	Aspergillus parasiticus	1.00E-23
Asp442	0.883	1-28(28)	hypothetical protein AN4235.2	EAA59334	Aspergillus nidulans	2.00E-76
Asp3646	0.567	1-26(26)	hypothetical protein AN8959.2	EAA63754	Aspergillus nidulans	1.00E-54
Asp1335	0.816	1-20(20)	Weakly similar to allergen asp f 4	O60024	Aspergillus fumigatus	8.00E-24
Asp1704	0.775	1-30(30)	Weakly similar to alpha 1,6 mannosyltransferase	CAD91643	Yarrowia lipolytica	4.00E-12
Asp2463	0.671	1-16(16)	Weakly similar to alpha,alpha-trehalose-phosphate synthase	NP_594728	Schizosaccharomyces pombe	8.00E-09
Asp1044	0.827	1-28(28)	Weakly similar to alpha-1,3-mannosyltransferase	AAQ86764	Cryptococcus neoformans	1.00E-08
Asp1617	0.936	1-18(18)	Weakly similar to antifungal protein precursor	JC4564	Penicillium chrysogenum	1.00E-08
Asp1831	0.855	1-27(27)	Weakly similar to carboxypeptidase s1	AAK77166	Aspergillus oryzae	1.00E-66
Asp2152	0.748	1-19(19)	Weakly similar to cell wall biogenesis protein	P38248	Schizosaccharomyces pombe	6.00E-32
Asp1291	0.494	1-18(18)	Weakly similar to cell wall synthesis protein	BAC82548	Penicillium chrysogenum	1.00E-12
Asp1813	0.933	1-19(19)	hypothetical protein FG09869.1]	XP_390045	Gibberella zeae	1.00E-104
Asp1074	0.841	1-30(30)	Weakly similar to dha14-like major facilitator	AAF64435	Botryotinia fuckeliana	2.00E-71
Asp2201	0.918	1-19(19)	Weakly similar to exo-1,3-beta-		Kluyveromyces	6.00E-15

			glucanase/1,3-beta-d-glucan glucanohydrolase	Q12628	lactis	
Asp2149	0.908	1-18(18)	Weakly similar to extracellular matrix protein	AAR06609	Magnaporthe grisea	5.00E-06
Asp4115	0.504	1-36(36)	Weakly similar to gaba permease	CAB43936	Emericella nidulans	2.00E-33
Asp5193	0.873	1-24(24)	hypothetical protein AN5450.2	EAA62610	Aspergillus nidulans	1.00E-92
Asp1421	0.599	1-25(25)	hypothetical protein	XP_328691	Neurospora crassa	3.00E-65
Asp533	0.627	1-38(38)	Weakly similar to hypothetical protein an0379.2	EAA66478	Aspergillus nidulans	5.00E-25
Asp2108	0.846	1-18(18)	Weakly similar to hypothetical protein an1449.2	EAA64579	Aspergillus nidulans	7.00E-20
Asp507	0.712	1-16(16)	Weakly similar to hypothetical protein an2869.2	EAA63440	Aspergillus nidulans	1.00E-05
Asp1614	0.953	1-24(24)	Weakly similar to hypothetical protein an4117.2	EAA59378	Aspergillus nidulans	2.00E-25
Asp1571	0.887	1-22(22)	Weakly similar to hypothetical protein an4422.2	EAA60339	Aspergillus nidulans	2.00E-14
Asp152	0.964	1-19(19)	Weakly similar to hypothetical protein an5335.2	EAA62495	Aspergillus nidulans	2.00E-08
Asp2045	0.925	1-19(19)	Weakly similar to hypothetical protein an5357.2	EAA62517	Aspergillus nidulans	7.00E-07
Asp4046	0.831	1-23(23)	Weakly similar to hypothetical protein an7041.2	EAA61687	Aspergillus nidulans	3.00E-09
Asp3349	0.708	1-36(36)	Weakly similar to hypothetical protein an7454.2	EAA62034	Aspergillus nidulans	1.00E-28
Asp1927	0.871	1-19(19)	Weakly similar to hypothetical protein an7941.2	EAA59595	Aspergillus nidulans	7.00E-08
Asp4355	0.599	1-34(34)	Weakly similar to hypothetical protein an8309.2	EAA66932	Aspergillus nidulans	1.00E-18
Asp2144	0.905	1-18(18)	Weakly similar to hypothetical protein an9450.2	EAA66809	Aspergillus nidulans	3.00E-15
Asp218	0.91	1-19(19)	Weakly similar to hypothetical protein fg02113.1	XP_382289	Gibberella zeae	1.00E-09
Asp4428	0.668	1-41(41)	Weakly similar to hypothetical protein fg03074.1	XP_383250	Gibberella zeae	4.00E-34
Asp1310	0.894	1-17(17)	Weakly similar to hypothetical protein fg08071.1	XP_388247	Gibberella zeae	2.00E-12
Asp1430	0.815	1-17(17)	Weakly similar to hypothetical protein mg01732.4	EAA56081	Magnaporthe grisea	2.00E-32
Asp3146	0.485	1-19(19)	Weakly similar to integral membrane family protein	NP_568386	Arabidopsis thaliana	5.00E-12
Asp3056	0.763	1-32(32)	Weakly similar to nucleobase permease, putative	CAF32038	Aspergillus fumigatus	4.00E-46
Asp130	0.578	1-22(22)	Weakly similar to oxidoreductase, mmyg	NP_639849	Streptomyces coelicolor	3.00E-20
Asp1818	0.803	1-21(21)	Weakly similar to pathogenicity protein	AAD01641	Magnaporthe grisea	3.00E-42

Asp3230	0.667	1-17(17)	Weakly similar to phd-type zinc finger	NP_588450	Schizosaccharomyces pombe	1.00E-24
Asp1918	0.936	1-18(18)	Weakly similar to predicted protein	EAA62532	Aspergillus nidulans	9.00E-10
Asp805	0.728	1-19(19)	Weakly similar to predicted protein	EAA62570	Aspergillus nidulans	1.00E-18
Asp881	0.705	1-25(25)	Weakly similar to predicted protein	EAA61476	Aspergillus nidulans	3.00E-06
Asp1070	0.744	1-24(24)	Weakly similar to predicted protein	EAA54079	Magnaporthe grisea	7.00E-27
Asp1697	0.642	1-32(32)	Weakly similar to predicted protein	EAA55453	Magnaporthe grisea	1.00E-25
Asp4873	0.84	1-15(15)	Weakly similar to predicted protein	XP_322535	Neurospora crassa	7.00E-33
Asp3814	0.632	1-25(25)	Weakly similar to predicted protein	XP_323565	Neurospora crassa	2.00E-18
Asp3602	0.654	1-25(25)	Weakly similar to predicted protein	XP_332102	Neurospora crassa	1.00E-14
Asp3305	0.758	1-18(18)	Weakly similar to predicted protein	XP_326695	Neurospora crassa	2.00E-16
Asp1230	0.846	1-17(17)	Weakly similar to predicted protein	XP_329231	Neurospora crassa	3.00E-18
Asp1292	0.787	1-31(31)	Weakly similar to predicted protein	XP_323368	Neurospora crassa	2.00E-39
Asp1546	0.898	1-19(19)	Weakly similar to predicted protein	XP_330658	Neurospora crassa	8.00E-25
Asp1547	0.946	1-19(19)	Weakly similar to predicted protein	XP_323846	Neurospora crassa	1.00E-13
Asp1606	0.914	1-23(23)	Weakly similar to predicted protein	XP_326639	Neurospora crassa	6.00E-48
Asp1709	0.912	1-33(33)	Weakly similar to predicted protein	XP_324766	Neurospora crassa	2.00E-16
Asp1866	0.824	1-34(34)	Weakly similar to predicted protein	XP_323995	Neurospora crassa	4.00E-16
Asp2148	0.745	1-30(30)	Weakly similar to predicted protein	XP_327196	Neurospora crassa	9.00E-18
Asp4784	0.89	1-21(21)	Weakly similar to protein of unknown function, green fluorescent protein (gfp)-fusion protein localizes to the endoplasmic reticulum; msc1 mutants are defective in directing meiotic recombination events to homologous chromatids; Msc1p	NP_013578	Saccharomyces cerevisiae	9.00E-16
Asp2275	0.811	1-45(45)	hypothetical protein AN6136.2	EAA57922	Aspergillus nidulans	8.00E-76
Asp1236	0.949	1-18(18)	hypothetical protein AN5125.2	EAA62306	Aspergillus nidulans	2.00E-20
Asp1413	0.768	1-27(27)	Weakly similar to signal peptidase-complex component	CAD10392	Candida albicans	2.00E-13
Asp1531	0.873	1-26(26)	Weakly similar to spherulin 4		Physarum	7.00E-14

			precursor	P11113	polycephalum	
Asp4708	0.937	1-19(19)	Weakly similar to stcf_emeni probable sterigmatocystin biosynthesis p450 monooxygenase STCF (Cytochrome P450 60A2)	EAA61606	Aspergillus nidulans	5.00E-35
Asp564	0.862	1-19(19)	Weakly similar to stcs_emeni probable sterigmatocystin biosynthesis p450 monooxygenase STCS (Cytochrome P450 59)	EAA61596	Aspergillus nidulans	1.00E-18
Asp941	0.552	1-15(15)	Weakly similar to the authentic, non-tagged protein was localized to the mitochondria; Yer004wp	NP_010919	Saccharomyces cerevisiae	2.00E-24
Asp3922	0.906	1-23(23)	hypothetical protein FG05404.1	XP_385580	Gibberella zeae	2.00E-40
Asp611	0.734	1-18(18)	Weakly similar to transmembrane transporter liz1p.	NP_596430	Schizosaccharomyces pombe	6.00E-46
Asp2493	0.721	1-19(19)	Weakly similar to transposase	BAC54731	Aspergillus oryzae	2.00E-08
Asp777	0.547	1-27(27)	Weakly similar to dehydrogenases with different specificities	ZP_00184180	Exiguobacterium sp.	1.00E-14
Asp4102	0.76	1-40(40)	hypothetical protein AN8328.2	EAA66951	Aspergillus nidulans	9.00E-11
Asp4682	0.749	1-39(39)	Weakly similar to unnamed protein product	CAD60573	Podospora anserina	1.00E-09
Asp139	0.921	1-23(23)	Weakly similar to zinc transporter zip (40.5 kd) (5o194)	NP_506393	Caenorhabditis elegans	1.00E-17
Asp1463	0.933	1-15(15)	xyloglucanase 2	AAO20340	Aspergillus aculeatus	1.00E-100
Asp1943	0.924	1-22(22)	xylosidase	CAB06417	Aspergillus niger	0
