# Supplementary material for: Generation, annotation, and analysis of an extensive Aspergillus niger EST collection
Source: BMC Microbiol. 2006 Feb 2;6:7. doi: 10.1186/1471-2180-6-7 (PMC1434744; doi:10.1186/1471-2180-6-7)
Supplement: Additional File 4 — Putative secretory pathway proteins. This file is a table listing the A. niger unisequences that code for proteins that are predicted to function in the secretory pathway. For each unisequence the table includes the unisequence identifier (column designated "Contig"), the predicted function assigned as described for Additional file 3, the GenBank ID for the BLAST subject that provided the predicted function, the BLAST subject organism, the associated Expect value and BLAST score, and the number of identical residues over the number of amino acids in the alignment. [file 1471-2180-6-7-S4.pdf]

**Additional file 4** Putative secretory pathway proteins

| Contig | Predicted function[a] | ID | Organism | E value | BIT scores | Identities |
|---|---|---|---|---|---|---|
| Secretion-related ER chaperones, foldases | | | | | | |
| **Asp84** | BIPA - protein translocation, folding, quality control | Y08868 | *Aspergillus niger* | 1e-70 | 268 | 138/157 (87%) |
| **Asp177** | kexin - release of mature proteins from their proproteins by cleavage | CAB64692 | *Aspergillus niger* | 2e-78 | 295 | 148/183 (80%) |
| **Asp494** | Homologous to protein kinase - Component of the unfolded protein response pathway (UPR) | AAP92915 | *Hypocrea jecorina* | 2e-43 | 178 | 100/181 (55%) |
| **Asp662** | cyclophilin-like peptidyl prolyl cis-trans isomerase (cypB) - folding of proteins | AY005867 | *Aspergillus niger* | 4e-66 | 251 | 125/138 (90%) |
| **Asp734** | Protein disulfide-isomerase A (PDIA) | X98797 | *Aspergillus niger* | e-109 | 399 | 195/195 (100%) |
| **Asp1020** | tigA - disulfide bond formation and isomerization, folding of proteins | X98748 | *Aspergillus niger* | 0.0 | 671 | 336/356 (94%) |
| **Asp1559** | KAR2-homolog of the mammalian BiP/GRP78 gene-protein folding and translocation | AAA34714 | *Saccharomyces cerevisiae* | 2e-88 | 330 | 169/262 (64%) |
| **Asp1882** | calnexin - folding and control of glycoproteins | CAC82717 | *Aspergillus niger* | 0.0 | 903 | 446/514 (86%) |
| **Asp1902** | Protein disulfide-isomerase A (PDIA) | X98797 | *Aspergillus niger* | e-130 | 467 | 234/248 (94%) |
| **Asp2143** | HSP70- protein folding | AY360059 | *Trichophyton verrucosum* | 0.0 | 993 | 511/610 (83%) |
| **Asp2274** | Homologous to peptidyl-prolyl cis-trans isomerase; fk506-binding protein | NP_595257 | *Schizosaccharomyces pombe* | 6e-22 | 105 | 48/63 (76%) |
| **Asp2680** | Weakly similar to fk506-binding protein 2 precursor (peptidyl-prolyl cis-trans isomerase) (PPIase) (Rotamase) (FKBP-21) (NcFKBP22) | O60046 | *Neurospora crassa* | 1e-15 | 86.3 | 39/53 (73%) |
| **Asp4188** | PDI related protein A (prpA)- disulfide bond formation and isomerization, folding of proteins | AF095899 | *Aspergillus niger* | e-110 | 400 | 201/208 (96%) |
| **Asp4603** | Homologous to proteasome regulatory subunit 12 - eliminating missfolding proteins | AAB84057 | *Hypocrea jecorina* | 2e-48 | 196 | 96/132 (72%) |
| **Asp5123** | Similar to cyclophilin-type peptidyl-prolyl cis-trans isomerase protein | NP_587769 | *Schizosaccharomyces pombe* | 2e-31 | 140 | 71/152 (46%) |

| | | | | | | |
|---|---|---|---|---|---|---|
| **Genes involved in protein transport, protein targeting and vesicle-mediated transport** | | | | | | |
| **Asp70** | secretion related GTPase (SrgB) ,<br>YptA- ER-Golgi transport<br>YPT1_YEAST GTP-binding protein YPT1 (Protein YP2) | CAC17833<br>AAF63333<br>P01123 | *Aspergillus niger*<br>*Aspergillus niger var.awamori*<br>*Saccharomyces cerevisiae* | 2e-78<br>4e-78<br>8e-65 | 294<br>293<br>249 | 145/145 (100%)<br>144/145 (99%)<br>120/147 (81%) |
| **Asp117** | Vacuolar protein sorting 16 (hVPS16) | Q9H269 | *Homo sapiens* | 8e-34 | 149 | 84/186 (45%) |
| **Asp154** | Similar to gcs1/glo3/sps18 family zinc finger protein; arfgap gtp-ase activating protein | NP_594843 | *Schizosaccharomyces pombe* | 1e-41 | 174 | 130/393 (33%) |
| **Asp158** | Similar to protein that localized to COPII-coated vesicles, involved in vesicle formation and incorporation of specific secretory cargo | NP_011461 | *Saccharomyces cerevisiae* | 3e-41 | 169 | 79/131 (60%) |
| **Asp171** | probable clathrin assembly protein AP47 | CAD70726 | *Neurospora crassa* | 3e-49 | 199 | 100/116 (86%) |
| **Asp214** | Similar to exocyst complex 70 kda component | CAE85518 | *Neurospora crassa* | 9e-40 | 168 | 100/257 (38%) |
| **Asp276** | signal recognition particle (SRP) 54 kd protein homolog | Q00179 | *Aspergillus niger* | e-103 | 379 | 196/197 (99%) |
| **Asp290** | Similar to coatomer beta subunit | NP_595403 | *Schizosaccharomyces pombe* | 5e-53 | 213 | 117/195 (60%) |
| **Asp341** | YP51_YEAST GTP-binding protein YPT51/VPS21 | P36017 | *Saccharomyces cerevisiae* | 3e-49 | 197 | 96/125 (76%) |
| **Asp383** | Homologous to coatomer gamma-2 subunit | CAE76361 | *Neurospora crassa* | e-130 | 468 | 242/305 (79%) |
| **Asp444** | syntaxin homologue [aspergillus oryzae]<br>similar to S. cerevisiae SED5" | BAC79150 | *Aspergillus oryzae* | 9e-58 | 227 | 122/136 (89%) |
| **Asp457** | Similar to kdel (lys-asp-glu-leu) endoplasmic reticulum protein retention receptor 2 [Mus musculus] | NP_080117 | *Mus musculus* | 1e-66 | 255 | 127/212 (59%) |
| **Asp550** | Similar to t-snare of the plasma membrane; sec9p | NP_011523 | *Saccharomyces cerevisiae* | 6e-18 | 96.3 | 49/108 (45%) |
| **Asp581** | NsfA - putative vesicular transport and membrane fusion protein; sec18 homolog | AAF63332 | *Aspergillus niger* | e-125 | 451 | 232/232 (100%) |
| **Asp586** | Homologous to endoplasmic reticulum calcium transporter | CAE11789 | *Ustilago maydis* | e-116 | 423 | 215/333 (64%) |
| **Asp635** | Weakly similar to v-snare binding protein; uba domain; n terminal exon may be incorrect | NP_593222 | *Schizosaccharomyces pombe* | 1e-11 | 72.4 | 46/146 (31%) |
| **Asp641** | Similar to coatomer gamma-2 subunit | CAE76361 | *Neurospora crassa* | 6e-30 | 136 | 69/99 (69%) |
| **Asp757** | Similar to vesicle transport v-snare protein | NP_596668 | *Schizosaccharomyces pombe* | 4e-23 | 110 | 64/154 (41%) |
| **Asp915** | Homologous to coatomer alpha subunit | AAC18088 | *Aspergillus nidulans* | e-143 | 512 | 269/359 (74%) |
| **Asp953** | hypothetical protein AN1768.2 - intra-Golgi transport, secretion related | EAA63944 | *Aspergillus nidulans* | 5e-21 | 106 | 54/58 (93%) |

| | | | | | | |
|---|---|---|---|---|---|---|
| **Asp1003** | Homologous to adapter-related protein complex 1 beta 1 subunit (beta-adaptin 1) (Adaptor protein complex AP-1 beta-1 subunit) (Golgi adaptor HA1/AP1 adaptin beta subunit) (Clathrin assembly protein complex 1 beta large chain) | O35643 | *Mus musculus* | e-104 | 382 | 190/316 (60%) |
| **Asp1139** | Homologous to Gdi1p - regulates vesicle traffic in secretory pathway by regulating dissociation of GDP from Sec4/Ypt/rab family of GTP-binding proteins | NP_011062 | *Saccharomyces cerevisiae* | 7e-64 | 248 | 165/223 (73%) |
| **Asp1217** | small GTPase AvaA, conteining Rab subfamily of small GTPases domain | BAB88682 | *Aspergillus nidulans* | e-107 | 389 | 194/205 (94%), |
| **Asp1218** | Similar to coatomer protein complex subunit beta 2 | NP_001001940 | *Danio rerio* | e-103 | 380 | 187/368 (50%) |
| **Asp1274** | vpsa - involved in vacuolar protein sorting | BAB78398 | *Aspergillus nidulans* | 0.0 | 830 | 422/462 (91%) |
| **Asp1296** | Similar to translocation protein sec62 | Q99161 | *Yarrowia lipolytica* | 2e-10 | 70.1 | 38/88 (43%) |
| **Asp1361** | putative vacuolar protein; beta-catenin family | NP_595238 | *Schizosaccharomyces pombe* | 2e-62 | 243 | 123/168 (73%) |
| **Asp1389** | Similar to Gdi1p - Regulates vesicle traffic in secretory pathway by regulating dissociation of GDP from Sec4/Ypt/rab family of GTP-binding proteins | NP_011062 | *Saccharomyces cerevisiae* | 5e-59 | 231 | 115/193 (59%) |
| **Asp1390** | Similar to rer1 homolog - Golgi proteins involved in ER retention(RER) | NP_080671 | *Mus musculus* | 1e-33 | 145 | 70/115 (60%) |
| **Asp1452** | Defective in vacuolar protein sorting; homologous to mouse SKD1 and to human hVPS4; Vps4p | NP_015499 | *Saccharomyces cerevisiae* | 4e-41 | 172 | 97/233 (41%) |
| **Asp1484** | Similar to Pmr1p - involved in Ca2+ dependent processes such as protein sorting, processing, and degradation in the Golgi | NP_011348 | *Saccharomyces cerevisiae* | 1e-56 | 225 | 140/366 (38%) |
| **Asp1597** | Similar to ACR190Cp - Golgi to plasma membrane transport, exocytosis | NP_983592 | *Eremothecium gossypii* | 1e-56 | 225 | 128/391 (32%) |
| **Asp1610** | YP31_YEAST GTP-binding protein YPT31/YPT8 | P38555 | *Saccharomyces cerevisiae* | 1e-69 | 265 | 127/179 (70%) |
| **Asp1619** | Weakly similar to snare protein; nsyn1 encode syntaxin homologs (post-Golgi t-SNAREs); | AAF72704 | *Neurospora crassa* | 4e-33 | 145 | 89/289 (30%) |
| **Asp1630** | Homologous to coatmer delta subunit | NP_588336 | *Schizosaccharomyces pombe* | 6e-55 | 216 | 117/197 (59%) |
| **Asp1708** | YP52_YEAST GTP-binding protein YPT52 | P36018 | *Saccharomyces cerevisiae* | 6e-36 | 153 | 91/190 (47%) |
| **Asp1774** | Homologous to synaptobrevin-like v snare protein | NP_596561 | *Schizosaccharomyces* | 5e-28 | 126 | 61/98 (62%) |

| | | | | | | |
|---|---|---|---|---|---|---|
| | [schizosaccharomyces pombe] | | *pombe* | | | |
| **Asp1811** | arf_ajeca adp-ribosylation factor, fgsc a4 - may modulate vesicle budding and uncoating within the Golgi apparatus | EAA66244 | *Aspergillus nidulans* | 2e-99 | 363 | 179/183 (97%) |
| **Asp1824** | RAS protein, conteining Rab subfamily of small GTPases domain | CAC17832 | *Aspergillus niger* | 2e-27 | 124 | 71/201 (35%) |
| **Asp1866** | Weakly similar to Nce102p - involved in secretion of proteins that lack classical secretory signal sequences | NP_015475 | *Saccharomyces cerevisiae* | 2e-13 | 77.8 | 53/143 (37%) |
| **Asp1874** | Homologous to cytoplasmic protein involved in release of transport vesicles from the ER; Sec13p | NP_013309 | *Saccharomyces cerevisiae* | 2e-75 | 285 | 144/240 (60%) |
| **Asp1880** | Putative sec61 | AAQ72809 | *Aspergillus awamori* | e-148 | 528 | 269/269 (100%) |
| **Asp2016** | Cdc48p- microsomal protein of CDC48 family of ATPases; involved in secretion, peroxisome formation and gene expression | AAM08677 | *Aspergillus fumigatus* | 0.0 | 1466 | 750/820 (91%) |
| **Asp2214** | Similar to protein that forms a heterotrimeric complex with erp1, erp2p, and Emp24, member of the p24 family involved in endoplasmic reticulum to Golgi transport; Erv25p | NP_013701 | *Saccharomyces cerevisiae* | 5e-49 | 196 | 95/193 (49%) |
| **Asp2357** | Weakly similar to golgi-localized protein with homology to gamma-adaptin, interacts with and regulates Arf1p and Arf2p in a GTP-dependent manner in order to facilitate traffic through the late Golgi; Gga2p | NP_011976 | *Saccharomyces cerevisiae* | 9e-17 | 92.0 | 62/183 (33%) |
| **Asp2417** | ACR190Cp - Golgi to plasma membrane transport, exocytosis | NP_983592 | *Eremothecium gossypii* | 6e-14 | 83.6 | 51/128 (39%) |
| **Asp2572** | Weakly similar to yeast synaptobrevin (v-snare) homolog bos1 | NP_593539 | *Schizosaccharomyces pombe* | 1e-30 | 135 | 77/214 (35%) |
| **Asp2720** | Similar to adapter-related protein complex 3 beta 1 subunit (Beta3A-adaptin)Adaptor protein complex AP-3 beta-1 subunit) (AP-3complex beta-1 subunit) (Clathrin assembly protein complex 3 beta-1 large chain) | O00203 | *Homo sapiens* | 6e-34 | 150 | 88/191 (46%) |
| **Asp2742** | Secretory pathway ca2+-atpase | AAF37300 | *Aspergillus niger* | 1e-94 | 351 | 180/195 (92%) |
| **Asp2795** | myosin I myoA | A56511 | *Aspergillus nidulans* | 3e-18 | 98.2 | 51/60 (85%) |
| **Asp2825** | similar to ADP-ribosylation factor-like 2, contained ARF-like small GTPases domain | XP_394559 | *Apis mellifera* | 4e-27 | 121 | 54/79 (68%) |
| **Asp2835** | Similar to t snare complex subunit; syntaxin-like [schizosaccharomyces pombe] | NP_593832 | *Schizosaccharomyces pombe* | 1e-25 | 120 | 76/189 (40%) |
| **Asp2898** | Homologous to secretory pathway ca2+-atpase | AAF37300 | *Aspergillus niger* | 2e-36 | 158 | 78/79 (98%) |
| **Asp2906** | Similar to geranylgeranyl transferase type ii alpha subunit | O93829 | *Candida albicans* | 2e-39 | 166 | 89/210 (42%) |

| | | | | | | |
|---|---|---|---|---|---|---|
| | (type ii protein geranyl-geranyltransferase alpha subunit) (GGTase-II-alpha) (PGGT) (YPT1/SEC4 proteins geranylgeranyltransferase alpha subunit) | | | | | |
| **Asp2931** | hypothetical protein AN6627.2 - SRP-dependent cotranslational membrane targeting, secretion related | EAA58156 | *Aspergillus nidulans* | e-105 | 385 | 200/310 (64%) |
| **Asp2935** | Weakly similar to SEC14 cytosolic factor (Phosphatidylinositol/phosphatidylcholine transfer protein) (PI/PC TP). | P24280 | *Saccharomyces cerevisiae* | 8e-14 | 80.9 | 57/214 (26%) |
| **Asp3240** | Weakly similar to protein transport protein sec61-gamma subunit | NP_593061 | *Schizosaccharomyces pombe* | 1e-18 | 94.4 | 40/70 (57%) |
| **Asp3374** | Aspergillus niger srgb gene for a secretion related gtpase of the YPT/RAB family, exons 1-5 | AJ278659 | *Aspergillus niger* | 0.0 | 252 | 123/123 (100%) |
| **Asp3383** | related to dock180 protein, involved in vesicle-mediated transport | CAE75725 | *Neurospora crassa* | 8e-67 | 260 | 129/240 (53%) |
| **Asp3413** | Similar to vacuolar protein sorting-associated protein vps35 | O74552 | *Schizosaccharomyces pombe* | 1e-39 | 168 | 84/222 (37%) |
| **Asp3534** | Similar to protein that forms a heterotrimeric complex with erp2p, emp24p, and Erv25p; member, along with Emp24p and Erv25p, of the p24 family involved in ER to Golgi transport and localized to COPII-coated vesicles; Erp1p | NP_009402 | *Saccharomyces cerevisiae* | 9e-41 | 169 | 92/225 (40%) |
| **Asp3633** | Similar to protein transport protein sec73 | O13817 | *Schizosaccharomyces pombe* | 8e-31 | 139 | 77/227 (33%) |
| **Asp3645** | Similar to gap1 protein | CAA22816 | *Schizosaccharomyces pombe* | 3e-19 | 100 | 52/93 (55%) |
| **Asp3812** | Vacuolar protein sorting-associated protein VPS13 (Intracellular trafficking and secretion) | Q07878 | *Saccharomyces cerevisiae* | 2e-29 | 136 | 77/221 (34%) |
| **Asp3899** | Similar to rab protein; involved in endocytosis | NP_594796 | *Schizosaccharomyces pombe* | 6e-38 | 160 | 72/125 (57%) |
| **Asp3952** | Vacuolar protein sorting-associated protein 74 | Q06385 | *Saccharomyces cerevisiae* | 1e-23 | 114 | 49/136 (36%) |
| **Asp4222** | Similar to secretion related GTPase gtpase, (SrgA) | CAC17832 | *Aspergillus niger* | 3e-30 | 134 | 65/65 (100%) |
| **Asp4247** | Similar to related to secretion protein rsec8 | XP_323530 | *Neurospora crassa* | 1e-41 | 176 | 89/171 (52%) |
| **Asp4285** | coatomer beta' subunit (beta' coat protein); secretory pathway | NP_596811 | *Schizosaccharomyces pombe* | 7e-39 | 166 | 73/111 (65%) |
| **Asp4301** | SED5-binding protein 2 (SEC24-related protein 2) Component of the COPII coat, that covers ER-derived vesicles involved in transport from the endoplasmic reticulum to the Golgi apparatus. | P53953 | *Saccharomyces cerevisiae* | 6e-29 | 133 | 78/183 (42%) |

| | | | | | | |
|---|---|---|---|---|---|---|
| **Asp4377** | GTP-binding protein SARA (A.niger) | P52886 | *Aspergillus niger* | 1e-79 | 298 | 146/162 (90%) |
| **Asp4401** | Similar to endocytosis and cytoskeleton protein - Golgi to endosome transport, secretion related | NP_587759 | *Schizosaccharomyces pombe* | 9e-09 | 65.1 | 27/47 (57%) |
| **Asp4442** | Protein transport protein sec72 | Q9P7V5 | *Schizosaccharomyces pombe* | 5e-41 | 174 | 92/224 (41%) |
| **Asp4460** | Coatomer beta subunit (Beta-coat protein) (Beta-COP) | Q9UUF7 | *Schizosaccharomyces pombe* | 4e-48 | 197 | 89/155 (57%) |
| **Asp4991** | Homologous to yeast component of copii coat of er-golgi vesicles, sec24 | P40482 | *Schizosaccharomyces pombe* | 8e-59 | 232 | 115/236 (48%) |
| **Asp5114** | Secretion related GTPase gtpase, (SrgA) | CAC17832 | *Aspergillus niger* | 1e-64 | 248 | 122/122 (100%) |
| **Asp5188** | Similar to vesicular-fusion protein sec17 homolog | Q9P6A5 | *Neurospora crassa* | 7e-60 | 233 | 114/203 (56%) |
| | | | | | | |
| | | **Post-translational modification** | | | | |
| **Asp170** | Similar to alpha-1,2-mannosyltransferase; ktr3p | NP_009764 | *Saccharomyces cerevisiae* | 3e-24 | 116 | 63/130 (48%) |
| **Asp370** | Homologous to mannosyltransferase pmti - functional equivalence to Saccharomyces erevisiae PMT2 | AAP05785 | *Hypocrea jecorina* | 5e-54 | 216 | 108/172 (62%) |
| **Asp458** | Weakly similar to 1,6-mannosyltransferase; involved in the outer chain elongation of N-linked oligosaccharides | NP_594852 | *Schizosaccharomyces pombe* | 8e-06 | 52.4 | 33/80 (41%) |
| **Asp483** | Similar to class i alpha-mannosidase 1a | AAG48160 | *Aspergillus nidulans* | 1e-46 | 192 | 104/239 (43%) |
| **Asp521** | mannosyl-oligosaccharide 1,2-alpha-mannosidase (ec 3.2.1.113) precursor | S63701 | *Aspergillus phoenicis* | 5e-98 | 361 | 178/184 (96%) |
| **Asp1044** | Weakly similar to alpha-1,3-mannosyltransferase | AAQ86764 | *Cryptococcus neoformans* | 1e-08 | 62.0 | 45/137 (32%) |
| **Asp1109** | Similar to mnn9p | AAK40024 | *Pichia angusta* | 6e-20 | 101 | 52/117 (44%) |
| **Asp1117** | Similar to dolichol phosphate mannose synthase- has been postulated to regulate the Trichoderma reesei secretory pathway. | BAB40714 | *Aspergillus oryzae* | 6e-51 | 203 | 100/106 (94%) |
| **Asp1340** | oligosaccharyltransferase alpha subunit | AAK08631 | *Aspergillus niger* | 3e-99 | 365 | 187/205 (91%) |
| **Asp1344** | Similar to mannosyl-oligosaccharide 1,2-alpha-mannosidase (Man(9)-alpha-mannosidase) | Q8J0Q0 | *Candida albicans* | 3e-55 | 219 | 107/214 (50%) |
| **Asp1579** | mannosyl-oligosaccharide 1,2-alpha-mannosidase (ec 3.2.1.113) precursor | S63701 | *Aspergillus phoenicis* | 3e-93 | 345 | 165/167 (98%) |
| **Asp1594** | Similar to class i alpha-mannosidase 1a | AAG48160 | *Aspergillus nidulans* | e-137 | 493 | 275/534 (51%) |
| **Asp1704** | Weakly similar to alpha 1,6 mannosyltransferase | CAD91643 | *Yarrowia lipolytica* | 4e-12 | 73.6 | 48/161 (29%) |
| **Asp2232** | Similar to hypothetical protein AN9084.2, predicted | EAA61917 | *Magnaporthe grisea* | 3e-73 | 281 | 141/189 (74%) |

| | | | | | | |
|---|---|---|---|---|---|---|
| | mannosyl-oligosaccharide 1,2-alpha-mannosidase activity | | | | | |
| **Asp2308** | Homologous to udp-n-acetylglucosamine:dolichyl phosphate N-acetylglucosamine-1-phosphate transferase; GPT | AAL78196 | *Aspergillus niger* | 1e-20 | 104 | 52/70 (74%) |
| **Asp2552** | Homologous to glycosyl transferase, putative | CAF32096 | *Aspergillus fumigatus* | 4e-53 | 212 | 102/148 (68%) |
| **Asp2774** | sec53p - protein-ER targeting, secretion related phosphomannomutase | CAE52248 | *Saccharomyces cerevisiae* | 2e-36 | 154 | 70/87 (80%) |
| **Asp3170** | Weakly similar to mannosyltransferase | AAK54761 | *Paracoccidioides brasiliensis* | 1e-29 | 133 | 75/188 (39%) |
| **Asp3205** | Homologous to alpha-1, 2-mannosyltransferase [mips] | XP_326881 | *Neurospora crassa* | 5e-12 | 72.0 | 26/42 (61%) |
| **Asp3381** | Similar to alpha 1,6 mannosyltransferase | CAD91643 | *Yarrowia lipolytica* | 7e-67 | 257 | 119/209 (56%) |
| **Asp3530** | Weakly similar to possible mannosylphosphorylation protein mnn4 protein | CAF31981 | *Aspergillus fumigatus* | 5e-38 | 161 | 86/225 (38%) |
| **Asp3936** | dolichyl-phosphate beta-glucosyltransferase | T44819 | *Ajellomyces capsulatus* | 6e-80 | 301 | 145/169 (85%) |
| **Asp4380** | Similar to golgi mannosyltransferase complex subunit | CAE00652 | *Kluyveromyces lactis* | 8e-45 | 184 | 85/150 (56%) |
| **Asp4472** | Weakly similar to Pmt1p - transfers mannose residues from dolichyl phosphate-D-mannose to specific serine/threonine residues of proteins in the secretory pathway; acts in complex with Pmt2p | NP_010188 | *Saccharomyces cerevisiae* | 2e-25 | 121 | 56/176 (31%) |
| **Asp4503** | Similar to mannosyltransferase | NP_595123 | *Schizosaccharomyces pombe* | 2e-63 | 246 | 106/188 (56%) |
| **Asp4598** | Weakly similar to alpha 1,6 mannosyltransferase | CAD91643 | *Yarrowia lipolytica* | 1e-08 | 61.6 | 34/103 (33%) |

[a]- the function of genes were identified using GO annotation and BlastX to nr protein
